# On Determination Method for Resolution of Secondary Electron Images in Scanning Electron Microscopy

**DOI:** 10.1002/advs.202519630

**Published:** 2026-05-25

**Authors:** Tongfang Yang, Yanbo Zou, Zejun Ding

**Affiliations:** ^1^ Hefei National Research Center for Physical Sciences at the Microscale and Department of Physics University of Science and Technology of China Hefei Anhui P. R. China; ^2^ School of Physics & Electronic Engineering Xinjiang Normal University Urumqi Xinjiang P. R. China

**Keywords:** Monte Carlo, Rayleigh criterion, resolution, Rose criterion, scanning electron microscopy, sharpness

## Abstract

A rational scheme for the determination of the resolving power of a scanning electron microscope (SEM) has been a long‐standing unresolved scientific issue. The objective measurement of the image resolution according to the ISO terminology definition is always difficult, and different measurement methods used in practice now are all inconsistent with the ISO terminology definition and do not follow the Rayleigh criterion. Here, we establish a sharpness‐resolution conversion curve method based on physical modeling of SEM imaging with the help of Monte Carlo simulation. The simulated secondary electron images of the resolution sample enable the build‐up of the conversion curve via the simultaneous evaluation of image sharpness and image resolution under both the Rayleigh criterion and the Rose criterion. The obtained conversion curves for realistic 3D structures of resolution samples show nonlinearity and provide a convenient means by programmed measurement routine, establishing a scientifically sound way and robust framework for automatic evaluation of SEM instruments based on their resolving performance.

## Graphical abstract

An idealized SEM, termed Rayleigh's microscope, is constructed by Monte Carlo simulation to represent imaging conditions that just satisfy the Rayleigh criterion. Based on this physically defined model, sharpness–resolution conversion curves are established and combined with the Rose criterion, enabling automated resolution evaluation from practical secondary‐electron images while clarifying the distinction between sharpness and resolution.

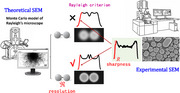

## 1 Introduction

Scanning electron microscopy (SEM) [1, 2] has played a vital role in the realm of material science, biology, nanotechnology, etc., offering an unparalleled view into the micro and nano‐scale architectures of materials. Since its inception, SEM has revolutionized our ability to characterize the morphology, dimension, and composition of diverse materials with increased resolving power. The resolution [3] of a magnified image is crucial to the characterization and, hence, the key metric for the specification of an SEM instrument. However, while high resolution is the hallmark of SEM quality, the resolution itself is a subtle, multifaceted metric in microscopy, and its accurate quantification is still challenging. This issue has been addressed in some of the previous publications. For example, Aert et al. pointed out that “resolution has always been, and still is, an important issue” [4] that is not unambiguously defined. Vladár et al. summarized that “Yet, it is one of the most elusive and least understood parameters”, “Determining the resolution achievable with a SEM is an elusive task. There are several definitions of resolution and a number of ways of calculating it. Only some of the methods are repeatable, and none of them is accurate or traceable” [5]. An International Organization for Standardization (ISO) document has indicated that “Even if various SEM manufacturers use the terms, the notion of resolution is not established scientifically, it is sample‐ and method‐dependent, and there is no accurate way of measuring it today” [6]. The present work then attempts to provide a scientific solution and a practical tool for this long‐standing problem.

Resolution of an SEM image has been defined in different ways in the literature [7]: (1) The minimum spacing. According to the ISO terminology definition of SEM the image resolution is defined as the “minimum spacing at which two features of the image can be recognized as distinct and separate” [8]. A direct approach to the implementation (the gap method) based on this definition is the measurement of the gap between two very fine particles or structures [5, 9, 10], which was commonly adopted many years ago by SEM manufacturers. However, the previously employed gap method relies on the human sense of the minimum resolvable gap [6], raising concerns about its accuracy and objectiveness. It also requires the quality on the resolution sample of particles on a substrate for a suitable particle density. On the other hand, in nature, the Rayleigh criterion of resolution described in literature [7] has a close relationship with the ISO terminology definition. The Rayleigh criterion [11, 12] is a classic method in optics to determine whether two bright spots observed by an optical instrument are distinguishable: when the first dark ring of one Airy disk for one diffracted bright spot coincides with the center of another Airy disk, the two spots are regarded as just distinguishable, and the distance between the centers of the tiny spots is the resolution. But, this Rayleigh criterion is difficult to employ in SEM: The particles under observation are usually large in size, and it is difficult to find the tiny particle pairs that just satisfy the criterion. Because the particles cannot be regarded as tiny spots in optics so that the distance between particle centers is not the resolution. In addition, the intensity profile of a particle in SEM imaging does not follow that of the Airy disk in optics. (2) Radial distribution or the point spread function (PSF). Some authors have proposed to measure the electron beam size [7, 13, 14] because it is regarded as the dominant factor to influence the image resolution; the resolution is defined as the width of the electron beam profile or a fraction thereof. But the measurement of the beam size [15] is usually very difficult and not suitable for a routine operation. Moreover, SEM resolution is not determined solely by the electron beam size, but is also influenced by multiple factors involved in electron‐solid interactions, the signal quality and components [16], etc. Therefore, the beam size alone cannot directly represent the ISO‐defined resolution. (3) Image sharpness. The concept of image sharpness in SEM was initially suggested from the human eye observation of micrograph quality, then an objective evaluation was proposed for quantifying high‐frequency components of Fourier transformation [17, 18, 19]. For a resolution sample, it is now evaluated from the gradient of edge intensity profiles of particles, and the ISO/TS 24597 standard [10] has established a programmed procedure for the evaluation of the image sharpness via three different ways to realize a practical objective determination. This definition has been commonly adopted by SEM manufacturers nowadays for the “resolution” evaluation, where the nominated “resolution” value (in fact, the sharpness) is measured by the statistical distance between two specified threshold values in the intensity profile of a particle edge. However, it must be emphasized that this definition of “resolution” for SEM performance evaluation does not follow exactly the ISO terminology definition of resolution, as we will clarify this concept later in this article, though both in literature and practice they are often conflated. (4) Edge resolution [7]. The definition is closely related to the beam diameter measurement and applies to the detection of the beam current transmitted through a sharp edge, while its quantification as the distance between two specified threshold values in the intensity profile is highly similar to the sharpness determination. (5) Spatial frequency. “Resolution” or sharpness is calculated based on the spatial frequency [20, 21, 22, 23] obtained from the Fourier transform of the SEM image. The ISO/TS 24597 standard [10] has also established a programmed procedure for the sharpness measurement based on a Fourier transformation. However, this definition of resolution or sharpness also does not follow the ISO terminology definition of resolution. In summary, the present status is that “various SEM manufacturers use some kind of spatial resolution calculation methods for their instruments, even if the “resolution” is not defined in scientifically sound ways” [8]. One may thus understand the necessity for developing a scientifically cogent measurement method and practical algorithm that is, on the one hand, based on the ISO terminology definition and physical principles of electron beam interaction with solids, and on the other hand, free of human interference for an objective, routine, and automatic resolution evaluation. We will approach this goal by integrating the distinctive features of the methods associated with each method mentioned above based on the physical modeling of the SEM imaging, together with an additional statistical estimation of the noise factor.

The image sharpness for SEM performance evaluation was first proposed by Postek and Vladár [17], who also developed a measurement method [18, 19, 20]. In recent decades, several automatic calculation procedures for sharpness have been developed, including the Fourier transform (FT) method [21, 22, 23], the derivative (DR) method [24, 25], the contrast‐gradient (CG) method [26, 27], and the autocorrelation function method [28, 29]. The ISO/TS 24597 standard [10] recommends the use of the FT, CG, and DR methods, and their calculation processes are standardized. This standardization, together with its ongoing project has advanced the performance evaluation among different SEM manufacturers and instruments toward a standardized objective way. In fact, there is also no commonly acknowledged definition about the sharpness in SEM, whereas in ISO standard about the terminology of micrographics [30], it is defined as “the subjective visual sensation of the ability of photographic material to show a sharp line of demarcation between two adjacent areas of an image, of different densities”. In concept, it is about a measure of the gradient of the edge intensity profile, i.e., the image blur caused by the PSF, which corresponds to the impulse response of the imaging system to the ideal imaging signal. In the ISO/TS 24597 standard [10], it is quantitatively evaluated as $\sqrt{2}\sigma$ via the sharpness factor (2*σ*), by assuming that the particle image is a binary disk, the ideal particle edge intensity profile is a semi‐infinite step function (see 4.3.4), and the intensity profile of the blurred edge image by PSF can be fitted with an error function as convolution of the step function with the 1D Gaussian function of the standard deviation σ for PSF. Although the sharpness is conceptually related to the resolution, it is not equivalent to the resolution as defined by ISO terminology [8], and the two differ significantly. The actual particle has 3D structure, and the particle edge intensity profile is not step‐function‐like as limited by the physical mechanism of signal emission during electron beam interaction with particles on a substrate system. In particular, the shape of the actual signal intensity profile depends on the particle size (and also the sample properties) and beam energy, while the error function modeling does not vary with these physical factors. Moreover, the system response is 2D but not 1D. Therefore, the considered simple conversion from sharpness factor (2*σ*) to the sharpness [10] for resolution determination is a poor approximation (see 4.3.4 and Supplementary Methods). Moreover, there are several different adoptions of profile thresholds for sharpness definition in practice by different SEM manufacturers, and a more restrictive interval definition yields the better “resolution” (i.e., the smaller resolution value), while the Rayleigh criterion presents a unique value.

Owing to its correlation with physical factors about sample, electron beam, and signal detection, the determination of resolution is much more complex and difficult than that of sharpness. Nevertheless, the sharpness is also inherently related to the resolution; if the image formation is simplified to a linear system with a PSF [23], one can then measure the system blur (i.e., the sharpness) and relate it with the minimal resolvable distance (or more exactly, “the minimum spacing” by ISO definition) under the Rayleigh criterion (i.e., the resolution). The key in this process is to establish a reasonable relationship between the sharpness (*R*) and the resolution (R) values, which include all physical factors influencing the image quality in a fictitious “Rayleigh SEM”, a theoretical modeling of practical SEM imaging. This will be done by a Monte Carlo simulation of SEM images for the exact evaluation of particle intensity profiles of a 3D structure in a realistic resolution sample to guarantee that the relationship built has solid physical bases.

Over the past few decades, significant progress has been made in the Monte Carlo simulation methods in electron beam‐related techniques [31, 32, 33, 34, 35, 36, 37], and the application of the Monte Carlo methods to SEM imaging is becoming a crucial tool in the understanding and interpretation of SEM data [38, 39, 40, 41, 42]. Ding and Shimizu have theoretically explored the relationships between the SEM image contrast and the resolution in the gap method by using a Monte Carlo simulation [9]. Mao and Ding have employed the Monte Carlo method to obtain the simulated SEM images of gold particles on a carbon substrate and calculated the image resolution based on the gap method and the CG method [43]. Zhang et al. have constructed a realistic sample morphology and particle structures for an Au/C sample from an experimental SEM image with the finite element triangular mesh and performed simulations of realistic SEM images [40]. Ruan et al. have further evaluated the sharpness of the simulated SEM images by using the programmed FT, CG, and DR methods as specified in ISO/TS 24597 [10] and discussed the performance of each method under different experimental conditions by varying the manipulable experimental parameters within the Monte Carlo simulation [44]. In particular, the ISO 21466 document [42] provides a systematic methodology for evaluating critical dimensions by CD‐SEM and establishes standardized protocols for image acquisition and measurement method. The standard emphasizes the integration of Monte Carlo‐based modeling into SEM image interpretation, enabling a further quantitative application of our understanding of electron‐solid interactions to the resolution and dimensional accuracy. Chen et al. have employed the state‐of‐the‐art Monte Carlo model to derive comprehensive information about SE1 and SE2 signals to explore the mechanism of ultrahigh resolution SEM imaging [16]. These studies have thus laid the cornerstone for the present work on the SEM resolution determination method.

In this study, to adapt to the Rayleigh criterion we consider the widely used resolution sample, gold particles on a carbon substrate (Au/C sample) [16, 21]. In addition, to account for the effect of image noise in practical SEM imaging, the Rose criterion (SNR of at least 4 is needed to be able to distinguish image features, see Section 4.3.3 and Section S1.3) [45, 46] is also considered. We have performed Monte Carlo simulations for equally sized a pair of gold particles on a carbon substrate to obtain the simulated SEM images for an ideal electron beam with zero diameter. Subsequently, a series of experimental blurring factors, including beam diameter (or focus), astigmatism, vibration, and noise were introduced through post‐processing to mimic the experimental blurring of SEM image. By applying the aforementioned methodologies in conjunction with the Rayleigh criterion and the Rose criterion, one can derive the *R* − R conversion curves between image sharpness and resolution. The *R* − R conversion curves established in the “Rayleigh SEM” can be applied to a practical SEM image in the sense of “calibration curves” to return a resolution value after the SEM image sharpness is evaluated by the improved DR method (together with the FT method for a pretreatment), which is detailed in Section S1.1. The determined resolution does not depend on the choice of thresholds for sharpness evaluation. Therefore, it becomes clear that by integrating these conversion curves with a sharpness evaluation algorithm, accurate assessment of SEM image resolution can be achieved via a programmed routine, effectively addressing a longstanding challenge in the field of SEM. This approach not only holds a strong physical foundation but also operates as a fully automated computational workflow, eliminating human intervention.

## 2 Results and Discussion

**FIGURE 1:**
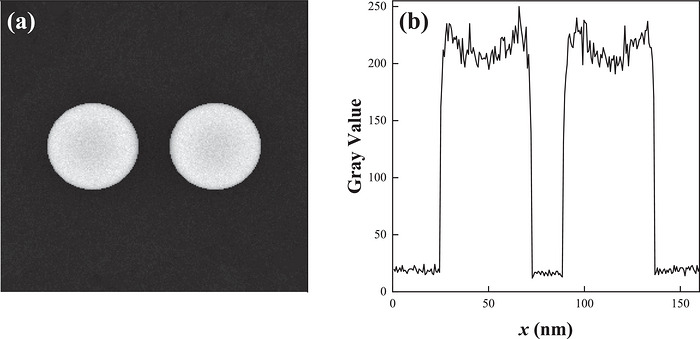
(a) Simulated SEM image of two gold spherical particles (diameter *d* = 30 nm) with a spacing *D* = 10 nm on a carbon substrate for an ideal beam of 1 keV. (b) Grayscale linescan profile along the line connecting centers of particles.

Figure 1a presents a simulated SEM image of two gold particles on a carbon substrate under an ideal experimental condition of the “Rayleigh SEM”, i.e., perfect beam (*σ* = 0) and the absence of vibration. The diameter of each gold particle is 30 nm, with a spacing of 10 nm between them. The simulated SEM image consists of 256 × 256 pixels, and for each pixel the trajectories of 10^3^ incident electrons and all cascaded secondary electrons in one‐two order greater are tracked. Electrons emitted from the material surface with energies less than 50 eV are directly counted as secondary electron signals for SEM imaging without the chance to come into a nearby particle, where the omission of the shadowing effect is valid for an in‐lens (and through‐the‐lens) electron detector. Their quantity determines the intensity, or gray value, of the corresponding pixels in the image. Figure 1b displays the corresponding grayscale linescan profile. As a result of the edge effect, the gray value is elevated at the edges of the gold particles.

**FIGURE 2:**
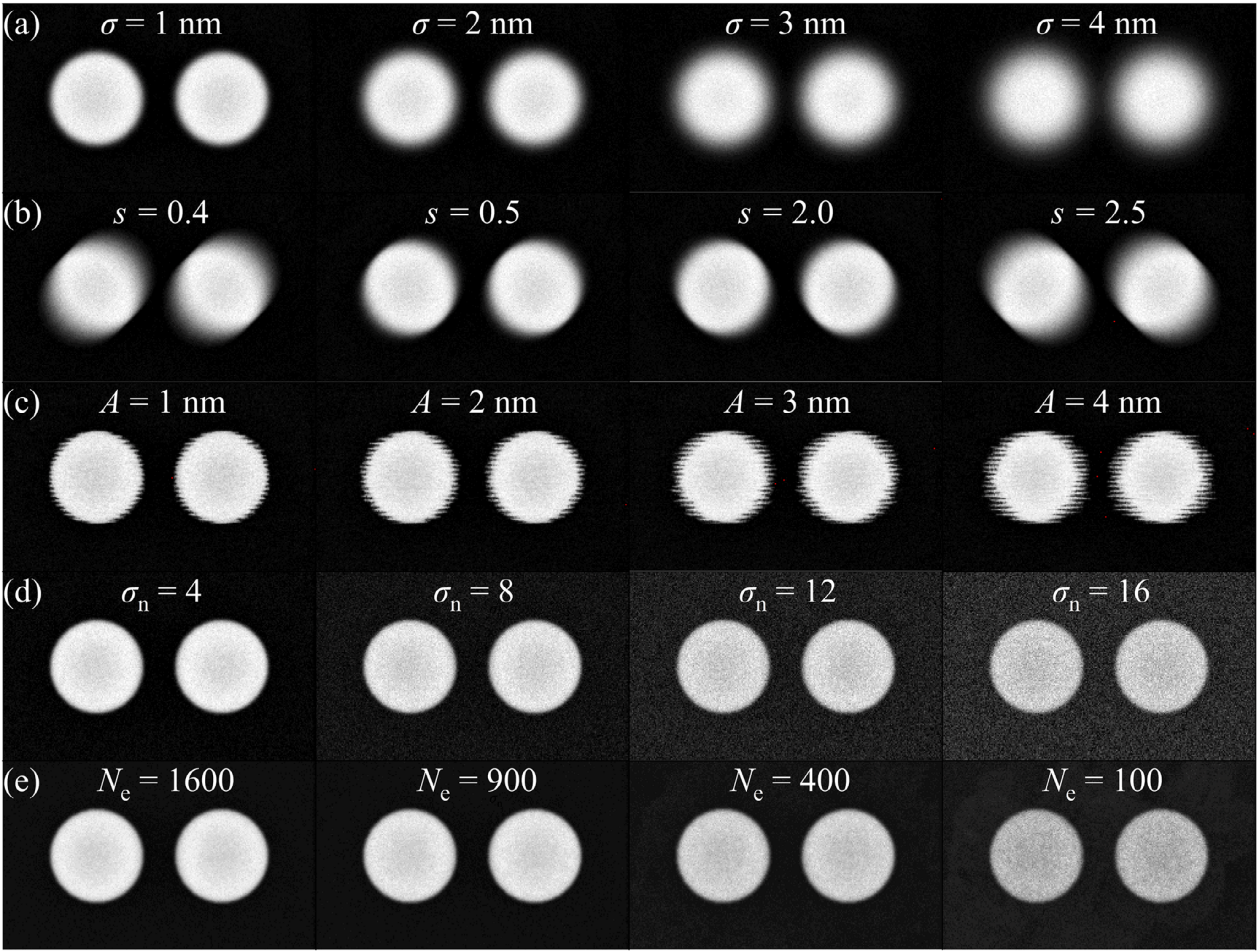
An overview of the impact of different experimental factors on simulated SEM image of two gold spherical particles (diameter *d* = 30 nm) with a spacing *D* = 10 nm on a carbon substrate for a beam of 1 keV by changing beam parameters: (a) focus σ = 1, 2, 3, and 4 nm; (b) astigmatism parameter *s* = 0.4, 0.5, 2.0 and 2.5 ($\varphi_{s}=45^{\circ }$ and *σ* = 1 nm); (c) vibration amplitude *A* = 1, 2, 3, and 4 nm (*ω* = 1 Hz and *σ* = 0.2 nm); (d) noise level *σ*_n_ = 4, 8, 12 and 16 (*N*_e_ = 10^3^ and *σ* = 0.5 nm); (e) number of incident electrons per pixel *N*_e_ = 1600, 900, 400, and 100 (*σ* = 0.5 nm).

Due to beam broadening, the signals collected for a pixel originate from a larger area nearby the incident location [16], resulting in a blurred image compared to the ideal scenario. The beam broadening can be described by a 2D Gaussian distribution for images in and near focus [23], and the convolution process, as a weighted averaging of the ideal image will remove the simulation noise in the initial image. The noise operation $F_{N}$ in Equations (13) and (14) is then applied to restore the original noise level. Figure 2a shows the simulated SEM images for different focus parameter *σ* values. As *σ* increases, the simulated particle image becomes progressively more blurred to enable calculation of sharpness and Rayleigh resolution. Hence, by varying spacing *D* value in the image simulation, one *R* − $R_{\mathrm{Rayleigh}}$ curve for a particular particle size (and shape) and beam energy can be built.

Figure 2b shows the simulated effect of astigmatism on the image blurring by Equations (9) and (10) for an astigmatism angle $\varphi_{s}=45^{\circ }$ and different astigmatism parameter *s* values. Astigmatism causes the electron probe to be stretched, leading to a corresponding stretching in the image. As *s* deviates from 1, the greater the degree of stretching and the poorer the image quality. The parameter settings of (*s*, *φ*_*s*_) and $\left(1/s,\varphi_{s}+90^{\circ }\right)$ are equivalent, resulting in mutually perpendicular stretching directions in Figure 2b. Figure 2c shows the simulated SEM images affected by vibration by Equations (11) and (12) for different vibration amplitudes. Vibration causes image distortion and jagged edges of particles; the degree of distortion increases with the vibration amplitude.

SEM images with varied noise levels (the standard deviation of the Gaussian noise) can be generated, as shown in Figure 2d, according to Equations (13) and (14), where the number of incident electrons *N*_e_ determining the Poisson noise is set in the figure as for the initial image, i.e., *N*_e_ = 10^3^. As noise increases, the contrast of the SEM image typically decreases. This occurs because noise introduces random signal fluctuations, increasing the randomness of the gray value and reducing the distinction between the effective signal and noise, which results in blurring of details and structures in the image. Additionally, the intensity of Poisson noise can be modified by changing the number of incident electrons *N*_e_, as shown by Figure 2e. The results differ somewhat from those in Figure 2d. When Gaussian noise is predominant, the entire image exhibits noise, while higher Poisson noise level is more concentrated in regions of higher gray values. These observations are consistent with the respective mechanisms of Poisson and Gaussian noise.

**FIGURE 3:**
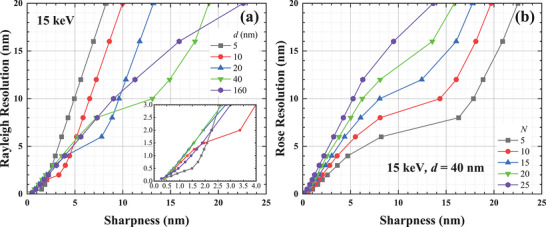
(a) Conversion curves for transforming sharpness *R* into Rayleigh resolution $R_{\mathrm{Rayleigh}}$ at an incident electron energy of 15 keV for varied particle diameter *d*; (b) Conversion curves for transforming sharpness *R* into Rose resolution $R_{\mathrm{Rose}}$ for varied noise level *N* at an incident electron energy of 15 keV and for a particle diameter of 40 nm.

Based on the methodological framework outlined above for integrating the Monte Carlo simulation method with the Rayleigh criterion, one can derive the sharpness‐resolution conversion curves depending on particle size and energy. Figure 3a presents an example of the *R–* $R_{\mathrm{Rayleigh}}$ conversion curves for a 15 keV beam and diameters of *d* = 5, 10, 20, 40, and 160 nm with particle shape specified in Figure S21. It is seen that the relationship between sharpness and Rayleigh resolution is not linear, particularly for large sharpness or resolution values. In ultrahigh resolution region, sharpness values of *R*_25*%* − 75*%*_ ∼ 1 − 2 nm correspond roughly to the resolution values of $R_{\mathrm{Rayleigh}}$ ∼ 0.7 − 2 nm (it is worth mentioning that the inset of Figure 3a shows that below 2 nm, the evaluated $R_{\mathrm{Rayleigh}}$ resolution value is smaller than the *R*_25*%* − 75*%*_ sharpness value), and in the low resolution region $R_{\mathrm{Rayleigh}}$ ≫ *R*, particularly for small particles. Likewise, combining the Monte Carlo method with the Rose criterion can yield the *R*–$R_{\mathrm{Rose}}$ conversion curves, which vary with particle diameter, energy, and noise intensity. Figure 3b displays an example of the *R* − ℜ_Rose_ conversion curves for 40 nm particles. $R_{\mathrm{Rose}}$ increases with noise level *N*. For small *N* (computed by Equation (18)), indeed $R_{\mathrm{Rose}}<R_{\mathrm{Rayleigh}}$ as expected, while at *N* ∼ 11.4, $R_{\mathrm{Rose}}=R_{\mathrm{Rayleigh}}$.

**FIGURE 4:**
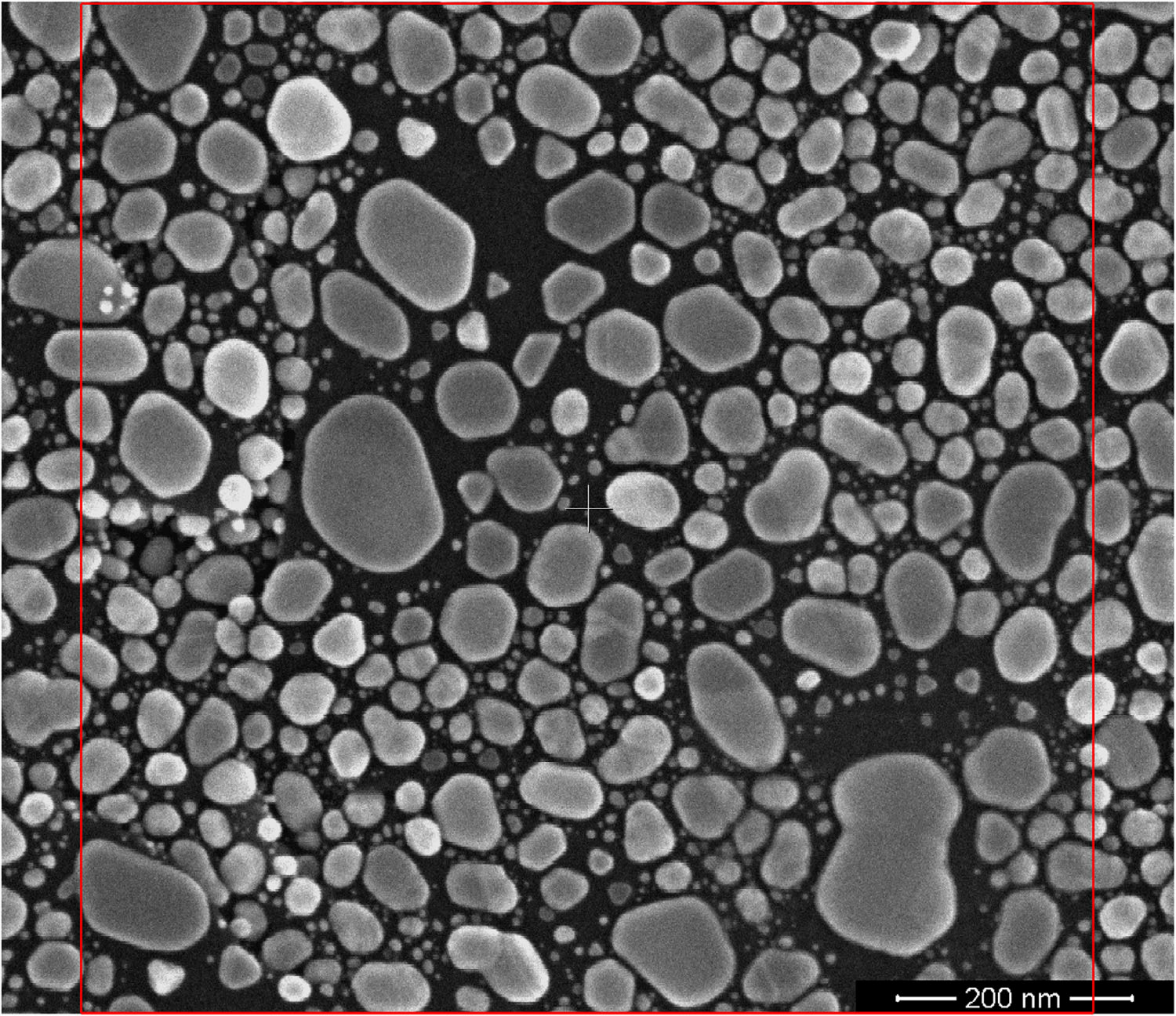
SEM image of an Au/C sample acquired at an incident electron energy of 15 keV. The red box indicates a 884 × 884 pixel region (without scale bar) used for analysis.

The present sharpness‐resolution conversion curve method has been tested for several SEM images of resolution samples (Au/C) obtained with different SEM instruments (see Section S4 for details). In the following, we present an example test and analysis results. Figure 4 shows an SEM image acquired at an accelerating voltage of 15 kV. A central region of 884 × 884 pixels (as indicated by the red box) is selected as the region of interest for evaluation. The sharpness is computed by using the present sharpness evaluation algorithm (i.e., the improved DR method), yielding a value of *R* ∼ 2.04  nm. The noise estimated is *N* ∼ 9.5. By applying the sharpness‐resolution conversion curves as shown in Figure 3, the Rayleigh resolution is evaluated as $R_{\mathrm{Rayleigh}}$ ∼ 1.99 nm, while the Rose resolution is $R_{\mathrm{Rose}}$ ∼ 1.64 nm. Therefore, the resolution ℜ value is determined as ℜ ∼ 2.0 nm. The calculated sharpness value is very close to the Rayleigh resolution. This is mainly because, for very narrow beam diameter ∼1 nm and for this resolution sample made mostly of the large particles, which dominate the amount of edge profiles, in sizes of several tens nm, the approximation of the geometrical model adopted for sharpness evaluation is good. For the measurement of the limit resolution of an SEM instrument, the image noise is usually quite low; therefore, only $R_{\mathrm{Rayleigh}}$ is effective. However, for the evaluation of daily performance SEM in service, $R_{\mathrm{Rose}}$ may become useful. When the sharpness algorithm is modified to use alternative edge definitions—e.g., *R*_15*%* − 85*%*_ or *R*_35*%* − 65*%*_ instead of the present *R*_25*%* − 75*%*_—the resulting sharpness value is changed to 3.14 nm or 1.17 nm, respectively. However, the final $R_{\mathrm{Rayleigh}}$ resolution value remained unchanged as 1.99 nm. This verifies that our resolution evaluation method does not depend on the specific sharpness metric choice in the intermediate step.

**FIGURE 5:**
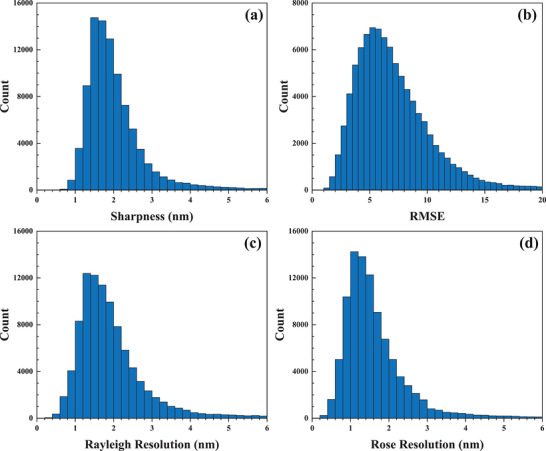
Distributions of (a) sharpness of edge intensity profiles derived from Figure 4, (b) RMSE values obtained from edge profile fitting, (c) Rayleigh resolution values, and (d) Rose resolution values converted from sharpness values of edge intensity profiles.

The measured sharpness of particles with different sizes in a practical SEM image—even when their visual edge sharpness looks similar—can vary when quantified by a calculation program. During the sharpness calculation process, a total number of 96,142 edge profiles were extracted from the image. The present improved DR algorithm has enabled the edge‐profiles extraction efficiency of ∼78% in comparison with ∼20% by the original algorithm [10]. The statistical distribution of the local sharpness values obtained from these edge profiles is shown in Figure 5a, exhibiting a peak around 1.6 nm and a rapid decline with increasing sharpness, and resulting in the mean value of sharpness *R* ∼ 2.04 nm. The sharpness is influenced by the particle size (and local shape as well as chemical composition), which agrees with practical experience. While the resolution of an SEM image (as a representation of an SEM instrument) should be a unique number, which is obtained by a statistical averaging over all particles, the determined value also relates partly to the properties of the resolution sample.

The fitting error associated with the curve fitting of edge intensity profiles for a local sharpness estimation was evaluated by using the root mean square error (RMSE), defined as

(1) $$\mathrm{RMSE}=\sqrt{\frac{1}{n}\sum_{i=1}^{n}{\left(I_{i}-{\hat{I}}_{i}\right)}^{2}}$$

where *I_i_* represents an extracted grayscale value in an edge intensity profile and ${\hat{I}}_{i}$ denotes the corresponding value in the fitted curve. The distribution of RMSE values corresponding to Figure 5a is presented in Figure 5b, where ∼84% of the cases exhibit RMSE values below 10 in comparison with the maximum gray value of 255, with the distribution peaking around 5.1.

Regarding resolution conversion efficiency, 97.9% of the extracted sharpness values were successfully transformed into Rayleigh resolution and Rose resolution values. The corresponding distributions of these values are shown in Figure 5c,d, respectively. The Rayleigh resolution distribution peaks at ∼1.4 nm with a mean of $R_{\mathrm{Rayleigh}}$ ∼ 1.99 nm, whereas the Rose resolution distribution peaks near 1.2 nm with a mean of $R_{\mathrm{Rose}}$ ∼ 1.64 nm.

**FIGURE 6:**
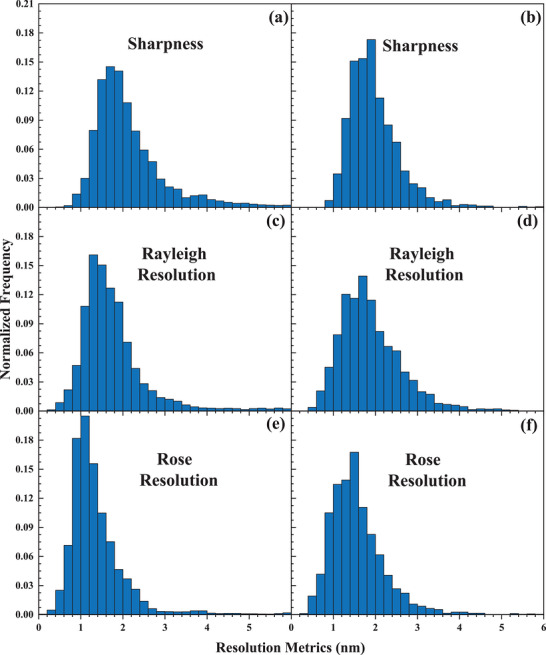
Normalized distributions of estimated values of (a,b) sharpness, (c,d) Rayleigh resolution, and (e,f) Rose resolution derived from edge profiles of particles with diameters ranging from 10–20 nm (left column) and 80–90 nm (right column) in the image of Figure 4.

Figure 6 compares the distributions of evaluated values of sharpness, Rayleigh resolution, and Rose resolution between small particles (10‐20 nm) and medium particles (80‐90 nm) from their edge profiles. It can be seen that the distributions have presented small variation of distribution shapes among different sizes. Bigger particles tend to present greater variance of resolution values perhaps due to the larger variation of local morphology. It is worth noting that the sharpness *R*_25*%* − 75*%*_ evaluated here differs from *R*_24*%* − 76*%*_ specified in ISO/TS 24597 [10] not only by the threshold definitions but mainly on the physical versus geometrical modeling. While the 3D morphology has not been addressed here, the *R* − R conversion curve method, however, should apply to any other type of resolution sample [47] and the associated morphologies. For the gold‐particle resolution sample, a well‐prepared sample with fully controlled particle shapes [48, 49] can be very useful to reduce the evaluation uncertainty; and if the particle shapes can be characterized prior to evaluation, the uncertainty is also expected to be reduced. The influence of noise level on the proposed resolution assessment scheme was also examined. With increasing noise level, the evaluated sharpness, Rayleigh resolution, and Rose resolution all increase, while the Rose resolution is much more sensitive to noise. This supports the use of the present hybrid strategy combining both the Rayleigh and the Rose criteria. Detailed procedures and analysis results are given in Supplementary Results.

In summary, this *R* − R conversion curve method integrates the ISO terminology definition of SEM image resolution, Rayleigh criterion and Rose criterion, modeling of SEM imaging based on the physical principles of beam‐sample interaction through a Monte Carlo simulation method, the principles of model‐based library method and the Shannon's rate‐distortion theory for information restoration of resolution value, the automatic sharpness calculation algorithm via FT and DR methods and the related ISO‐defined procedure into a unified framework for resolution determination.

## 3 Conclusions

This work presents a method for the determination of resolution of SEM images where the term resolution follows exactly the ISO‐defined terminology (i.e., the minimum spacing at which two features of the image can be recognized as distinct and separate), by applying the Rayleigh criterion and the Rose criterion. Definite differences between the concepts of image sharpness and resolution have been clarified, i.e., the estimated image sharpness only refers to the image quality while the evaluated resolution relates to physical factors about SEM instrument and sample. The conventional “resolution” measurement methods, in fact, deal with the image sharpness rather than the resolution; and standardized sharpness calculation algorithm (the DR method) in the ISO/TS 24597 document is based on a geometrical model with several assumptions. In particular, the assumption that the particle is a 1D semi‐infinite structure, such that the sharpness relates to beam broadening via a simple linear relationship is poor when applied to tiny nanoparticles of several nanometers. The realistic edge sharpness in practice still involves many physical factors about sample (chemical property, 3D morphology, and size of structure), electron beam (primary energy and beam width), and detector condition (type of detectors and detected signals, working distance, and extraction voltages), which influence image resolution through combined effects on signal properties. Hence, sharpness is inherently related to resolution, and the resolution value can be assessed from sharpness value once a physical modeling of SEM imaging (the “Rayleigh SEM”) is established. We first simulate a theoretical SEM image of a pair of equally sized gold particles on a carbon substrate in the fictitious “Rayleigh SEM”. Experimental beam parameters, such as focus, astigmatism, vibration, and noise can be added to the simulated SEM images to mimic the blurred experimental image through post‐processing. The sharpness is then measured by the thresholds of edge intensity profile as a compressed index of the intensity profile for information transmission. The sharpness and resolution values are calculated to derive a sharpness‐resolution conversion curve, where the important physical factors are incorporated into the conversion curve. The established *R* − R conversion curves enable one to convert the sharpness values of particle edges into a unique image resolution value that cannot be directly assessed by an experimental measurement method. This study then provides, in principle, an ideal scheme for the objective determination method of SEM resolution, a long‐standing unresolved scientific issue. The numerical algorithms presented in this work also offer a concrete and systematic approach for implementation of the *R* − R conversion curves.

## 4 Methods

### 4.1 Monte Carlo Model

A state‐of‐the‐art classical trajectory Monte Carlo simulation method is employed to simulate SEM images. This section outlines the Monte Carlo model because its details have been described elsewhere [37, 50]. The sample 3D structure is modeled by using a finite element triangular mesh. Next, the modeling of electron interaction with solids is described, covering the basic physical processes of electron elastic scattering, electron inelastic scattering, cascade secondary electron generation, and electron emission. During electron transport, elastic scattering causes the deflection of the electron moving direction, while inelastic scattering engenders change of electron velocity vector for both direction and magnitude, and the excitation of secondary electrons. After multiple scatterings the incident electrons and excited secondary electrons may be emitted from the sample surface to become backscattered electron (BSE) signals (>50 eV) or true secondary electron (SE) signals (<50 eV).

#### 4.1.1 Sample Structure Modeling

For a Monte Carlo simulation of SEM image of an arbitrary 3D sample [38, 39, 40, 41], a highly versatile finite element triangular mesh model [51] is employed in this work to represent the surface topography of sample. A large number of triangles are usually required to ensure that the modeled surface approximates well the actual sample. To reduce the computation time required for judging the intersections between electron trajectories and the triangles, a method involving spatial subdivision and indexing [40] was adopted. This approach divides the whole simulated space into uniformly sized small cubic segments. When sampling the next flight step of an electron trajectory, only those triangles within the final cube of the flight destination need to be considered, rather than testing all the triangles in the entire space. This method greatly simplifies the intersection determination process and significantly reduces computational load. The spatial subdivision and finite element mesh modeling techniques are detailed in our previous work [39, 40].

#### 4.1.2 Electron Elastic Scattering

In the present Monte Carlo simulation, we have employed the relativistic expression for electron elastic scattering due to the electron Coulomb's interaction with an atomic nucleus, or more specifically the Mott's differential cross‐section [52]:

(2) $$\frac{d\sigma_{e}}{d\Omega }={\left|f\left(\theta \right)\right|}^{2}+{\left|g\left(\theta \right)\right|}^{2}$$

where *θ* is the angle of electron scattering into the solid angle dΩ. The scattering amplitudes *f*(*θ*) and *g*(*θ*) can be calculated by using the partial‐wave expansion method [53]. In this work, the ELSEPA code [54] was used to obtain numerically the elastic scattering cross section. The Dirac‐Fock electron density [55] and the Fermi distribution were employed to calculate the electronic and nuclear charge densities, respectively. Additionally, the calculation also incorporated the correlation‐polarization potential, which is based on the local density approximation as well as the Furness‐McCarthy exchange potential [56].

#### 4.1.3 Electron Inelastic Scattering

To describe the inelastic interaction between a kinetic electron and a material, a dielectric response theory was employed. The differential inverse of inelastic mean free path (DIIMFP) is given as,

(3) d2λin−1dℏωdq=1πa0EIm−1εq,ω1q

where *λ*_in_ represents the electron inelastic mean free path or the inverse inelastic scattering cross section, *a*_0_ denotes the Bohr's radius and *E* is the electron kinetic energy. The complex dielectric function ε(*q*, ω) of the medium depends on the momentum transfer ℏ*q*, which is related to the scattering angle, and the energy loss ℏ*ω*. The energy loss function (ELF), Im{−1/ε(q,ω)}, thus plays a crucial role in determining the probability of inelastic scattering events. It quantifies the likelihood that an electron will lose energy to excite the electronic states of the material, thereby influencing the overall dynamical history of the moving electron. Due to the limited knowledge of the ELF for q≠0, several algorithms have been proposed to extend the experimental optical ELF data measured at the optical limit (q→0) into the general (*q*, ω)‐plane. All the information about the electronic states and excitation probabilities of a particular material are included in the measured optical ELF data. The full Penn algorithm [57] was employed in this work to achieve extension by using the Lindhard ELF [58] as expansion basis:

(4) Im−1εq,ω=∫∞0gωpIm−1εLq,ω;ωpdωp

where the expansion coefficient is given by the optical ELF as,

(5) gω=2πωIm−1ε0,ω

The Lindhard model for free electron gas incorporates electron‐electron interactions by assuming that each electron interacts with the average electric field generated by all other electrons, known as the random‐phase approximation. The Lindhard ELF consists of two terms arising from plasmon excitation and single‐electron excitation, which govern the different regions in the (*q*,*ω*)‐plane, and the algorithm employs distinct methods to calculate these components [59].

#### 4.1.4 Electron Cascading

An electron undergoing inelastic scattering will lose energy to the solid to excite electronic states and, hence, induce the generation of secondary electrons. These secondary electrons are produced within the Penn algorithm through two types of inelastic scattering [59]: plasmon excitation and single‐electron excitation. In the case of plasmon excitation, an electron with energy *E*′ in the Fermi sea gains the energy loss ℏ*ω* from a scattering electron and becomes the excited secondary electron with energy *E*′ + ℏω. The probability distribution of *E*′ is proportional to the joint density of states of free electrons, denoted as $p\left(E^{\prime },\omega \right)\propto \sqrt{E^{\prime }\left(E^{\prime }+\hbar \omega \right)}$. For single‐electron excitation, the secondary electron is excited in a disk‐shaped region within the Fermi sphere, as determined by the conservation laws of energy and momentum. The probability distribution of the initial state is described by [60]:

(6) $$\begin{array}{ccc} p\left(\left|k_{i}\right|<k_{F};q,\omega \right) & = & \int dk_{i}\delta \left[\hbar \omega -\frac{\hbar^{2}}{2m}\left(2k_{i}\cdot q+q^{2}\right)\right]\\ & & \times \;\Theta \left(kF-\left|k_{i}\right|\right)\Theta \left(\left|kf\right|-kF\right) \end{array}$$

where ***k***_i_ is the initial wave vector of the electron, ***k***_f_ is the Fermi wave vector, and ***k***_f_ = ***k***_i_ + ***q*** is the wave vector of the excited secondary electron. An excited secondary electron will undergo similar elastic scattering and inelastic scattering and generate further the lower energy secondary electrons; thus, an incident kinetic electron can produce many low‐energy secondary electrons inside the solid in a cascade process [50].

#### 4.1.5 Electron Emission

After multiple scattering events, an electron moving into the surface region can have a chance for emission from the sample surface into vacuum if the kinetic energy is enough to overcome the surface barrier. According to quantum mechanics, the probability of electron emission is given by the transmission function [61],

(7) $$T\left(E,\beta \right)=\left\{\begin{array}{cc} \frac{4\sqrt{1-U_{0}/E\cos^{2}\beta }}{{\left[1+\sqrt{1-U_{0}/E\cos^{2}\beta }\right]}^{2}}, & \mathrm{if}E\cos^{2}\beta >U_{0};\\ 0, & \mathrm{otherwise} \end{array}\right.$$

where the potential barrier *U*_0_ is the sum of Fermi energy and work function, *β* is the angle between the electron's moving direction and the surface normal. When an electron is successfully emitted from the sample surface, its kinetic energy relative to the vacuum level decreases by *U*_0_. Statistically only those secondary electrons generated near the surface region (<1 nm) [62] can have a chance to be emitted from the surface to be the true SE signals for imaging. BSE signals are also collected to estimate SE3 signals detected by an Everhart‐Thornley detector, as presented in Section S4.

### 4.2 Image Blurring Model

The ideal SEM image *I*_o_ was first simulated for an electron beam at zero diameter incident onto the resolution sample made of a pair of gold particles on carbon substrate by varying particle diameter and the spacing between the two particles. Then the system response functions were imposed on the ideal image to consider experimental factors and to model the blurred images under experimental observations. In our previous work [44], some experimental factors were incorporated directly in the Monte Carlo program. In this study, the ideal image was post‐processed with some key factors to model different experimental conditions.

#### 4.2.1 Focus and Astigmatism

The most important blurring factor for image degradation is due to the broadening of the primary electron beam at the local surface of the sample. Usually, the cross‐sectional profile or the PSF is represented by a Gaussian distribution, while in reality the beam shape varies with the extent of electron diffraction and lens aberrations [10]. Here, we adopt the same approximation of Gaussian distribution as employed in ISO/TS 24597 [10]. By performing 2D convolution on the ideal image *I_o_*, a series of blurred images with varied degrees of electron beam focus and astigmatism can be obtained:

(8) $$I_{f}\left(i,j\right)=\sum_{x=-m}^{m}\sum_{y=-n}^{n}I_{o}\left(i-x,j-y\right)g\left(x,y\right)$$

where *i* and *j* represent the horizontal‐ and vertical‐position of a pixel in the image, respectively. *m* and *n* determine the range of the 2D convolution. Considering the beam profile stretch due to astigmatism, the convolution kernel *g*(*x*, *y*) taken as a 2D Gaussian distribution has the form [63],

(9)

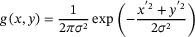

(10) $$x^{\prime }=s\left(x\cos\varphi_{s}+y\sin\varphi_{s}\right),\;y^{\prime }=\frac{1}{s}\left(-x\sin\varphi_{s}+y\cos\varphi_{s}\right)$$

where *σ* is the standard deviation of the 2D Gaussian distribution, referred to in this article as the focus parameter. *s* serves as the astigmatism parameter, indicating the extent of astigmatism, and *φ*_*s*_ represents the rotation of the astigmatism, the astigmatism angle.

#### 4.2.2 Vibration

The SEM device is inevitably affected by the surrounding environment, leading to tiny mechanical vibrations of the instrument, which manifest in the SEM images as small horizontal burrs or distortions at the particle edges. An image affected by vibrations can be generated by superimposing the initial image:

(11) $$I_{v}\left(i,j\right)=\frac{1}{n}\sum_{k=1}^{n}I_{o}\left(i+\Delta x\left(t_{k}\right),j\right)$$

where the summation *k* spans the total duration of all moments in a pixel imaging cycle. Δ*x*(*t*) represents the displacement caused by the vibration at time *t*, expressed as a superposition of sine functions [63]:

(12) $$\Delta x\left(t\right)=\sum_{i}A_{i}\sin\left(\omega_{i}t+\psi_{i}\right)$$

where the amplitude *A* is randomly sampled from a Gaussian distribution, *ω* is a given vibration frequency, and *ψ* is a randomly selected phase shift.

#### 4.2.3 Noise

The original images generated by a Monte Carlo simulation contain certain statistical noise due to the limited number of simulated electron trajectories; however, the aforementioned convolution operation to model the beam focus factor removes this noise. To quantify the noise influence on the resolution under Rose criterion, we reintroduce two types of noise into the SEM images: Poisson noise [64] and Gaussian noise.

The presence of Poisson noise in SEM images arises from the particle nature of electrons. Electron signals arrive at a detector discretely and randomly, following a Poisson process. The gray value of each pixel is proportional to the number of electron signals collected by the detector, resulting in an error distribution that follows a Poisson distribution. As the Poisson distribution approximates a normal distribution when a large number of particles are collected, the noise distribution is typically close to the normal distribution under common conditions. In our simulation, the number of electrons *N* detected per pixel is randomly sampled according to a Poisson distribution, with a mean of *N*_e_, as follows:

(13) $$P\left(N=k\right)=\frac{e^{-N_{e}}N_{e}^{k}}{k!}$$

where 𝑘 denotes the actual number of electrons received by the detector for a given pixel. Gaussian noise originates from various electronic components of the SEM imaging system, including detectors, amplifiers and other electronic circuitry. The Gaussian distribution represents the combined effect of numerous small, random fluctuations in the electronic signals. In our simulation, Gaussian noise is implemented using the following formula:

(14) $$I_{g}=I_{o}+R_{n}$$

where *R*_n_ is a random number following a Gaussian distribution with a mean of zero, its standard deviation σ_n_ determines the intensity of the Gaussian noise.

### 4.3 Sharpness and Resolution Determination

In conventional optical imaging, the object is typically described by an object function *I*_o_(*x*), which reflects the spatial variation of a physical property on the object plane. The response of the imaging system is mainly characterized by its PSF, which defines the spatial distribution of a single point from the object in the recorded image. In linear imaging systems, the final image *I*(*x*) is formed by the cumulative contributions of all object points, each modulated by the PSF. Mathematically, this process is expressed as the convolution of the object function with the system PSF, i.e., $I\left(x\right)=I_{o}\left(x\right)⊗\mathrm{PSF}\left(x\right)$. Similarly, in the case of SEM, the image intensity *I*(*x*) is represented by the system response (the function F as a math operation) of object function *I*_o_(*x*) as,

(15) $$I\left(x\right)=F\left\{I_{o}\left(x\right)\right\}$$

where *I*_o_(*x*) is an ideal image; hereafter, the phrase “ideal” represents the hypothetical case of an infinitely narrow electron beam. The response F includes various factors blurring the ideal image. The distinctive concepts of sharpness and resolution can be distinguished by the approximations leading to *I*_o_(*x*) and F.

#### 4.3.1 Sharpness

**FIGURE 7:**
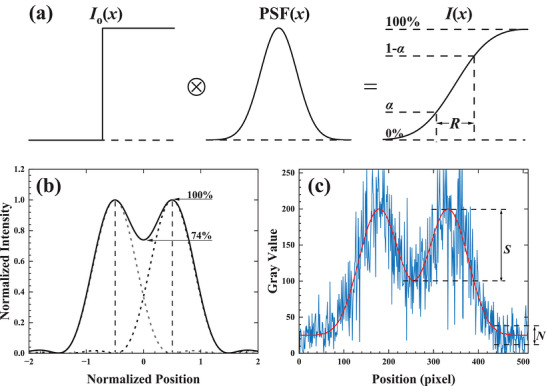
(a) Schematic diagram for the convolution process in the sharpness evaluation from a curve shape fitting by a geometrical model, where *α* and 1 − *α* are the lower and higher thresholds for the definition of sharpness. The present method adopts the 25%–75% thresholds; (b) Intensity distribution of two overlapping Airy disks in optics just satisfying the Rayleigh criterion; (c) Schematic diagram of the SEM linescan profile (blue line) of two adjacent particles when their separation is sufficiently large, such that the Rose criterion, S/N≥4, is satisfied.

The standardized sharpness calculation algorithm (the DR method) [10] is based on several rough assumptions that: 1) the ideal SEM image of particles can be considered as binary; 2) the ideal image function at the particle edge can be taken as a 1D step function (Heaviside function), $I_{o}\left(x\right)=I_{\Theta }\equiv \Theta \left(x\right)$, where Θ(x){=0,x<0;=1,x>0}, for a semi‐infinite 1D structure; 3) the system response F is the 1D PSF. Therefore,

(16) $$I\left(x\right)=I_{\Theta }\left(x\right)⊗\mathrm{PSF}\left(x\right)$$

where PSF(*x*) is approximated as a Gaussian function, $G\left(x;\sigma \right)=\exp\left(-x^{2}/2\sigma^{2}\right)/\sqrt{2\pi \sigma^{2}}$ and the standard deviation *σ* characterizes the spatial broadening of the beam profile and can therefore be used as a parameter describing the focusing condition or the effective beam diameter. As shown in Figure 7a, the intensity profile *I*(*x*) at an edge thus takes the form of an error function:

(17) $$I\left(x\right)=\frac{1}{2}+\frac{1}{2}\mathrm{erf}\left(\frac{x}{\sigma \sqrt{2}}\right)$$

In ISO/TS 24597 [10] for evaluation of a practical SEM image, the algorithm of the DR method extracts edge intensity profiles from particles in an SEM image and fits them with error functions by using Equation (17). The sharpness value is derived from a statistical averaging of *σ* corresponding to an error function for all extracted profiles as $R_{24\%-76\%}=\sqrt{2}\sigma$, as it was assumed to relate to Rayleigh criterion sharpness/resolution [10].

But, the above approximations are generally poor for sharpness evaluation, let alone for resolution evaluation. This is because, even for such a “geometrical model” by neglecting the physical factors involved in electron beam interaction with a sample, the particles in a resolution sample cannot be considered as 1D semi‐infinite structure (except when beam diameter is extremely small), and the PSF of imaging system is not 1D Gaussian function. Therefore, the sharpness (and resolution) should involve the factor of particle size, and the general metric $R_{24\%-76\%}=\sqrt{2}\sigma$ is simply an approximation.

#### 4.3.2 Rayleigh Criterion

More essentially, the sharpness is not equivalent to the resolution defined by the ISO terminology [8], both in concept and number. For an ideal electron beam of zero diameter, the ideal particle image *I*_o_(*x*) still shows an intensity distribution that varies with particle size, shape, and beam energy, results from physical principles involved in electron beam interaction with the sample. Moreover, the real sample structure is 3D, and a 2D PSF should be considered (even for the geometrical model as described in Section S1.2). In this study, the ISO terminology definition of SEM image resolution, more specifically the minimum resolvable spacing, is adopted for the resolution assessment. To establish a quantitative relationship between image sharpness and resolution, we will combine the use of the Rayleigh criterion and the Rose criterion on the Monte Carlo simulated SEM images of the resolution sample. Within this physical model, we take $I_{o}\left(x,y\right)=I_{\mathrm{MC}}\left(x,y\right)$, and F=PSF for Rayleigh criterion and $F=\mathrm{PSF}\cdot F_{v}\cdot F_{N}$ for Rose criterion, where PSF = *G*(*x*, *y*; *σ*), $F_{v}$ is the vibration operation (Equation (11)) and $F_{N}$ is the noise operation (Equations (13) and (14)).

The Rayleigh criterion [12], originated from classical optics, states that when the first‐order dark ring of one Airy disk (the image of a light spot) coincides with the center of another Airy disk, the two disks are just distinguishable, as shown in Figure 7b. In this scenario, the depth of the trough between the two particles is 74% of the intensity maximum. In an SEM image of a small particle, the intensity distribution shape is like that of the zero‐order diffraction spot of the Airy disk, as shown by Figure S4c (while in the general case a large particle presents intensity closer to Figure S4f). Thus, the same number of 74% is employed for the Rayleigh resolution criterion (whereas 60% is implied in sharpness evaluation [10]). Here, a 2D PSF(*x*, *y*) is responsible for the image blur due to electron beam focus or the beam broadening.

#### 4.3.3 Rose Criterion

Practical images inevitably contain certain signal noise, which influences the image quality and resolution [65], while the Rayleigh criterion leaves this issue out of consideration. The DR method extracts edge intensity profiles from particles in an SEM image and fits them with smooth error functions by either the original DR algorithm [10] or the present improved DR algorithm. This processing thus implies the image noise is actually ignored (once the required contrast to gradient level [10] meets). The Rose criterion gives an alternative resolution quantification criterion for optical imaging systems based on the signal‐to‐noise ratio (SNR). Its core definition states that two adjacent signal sources can be reliably resolved if their combined intensity distribution yields an SNR of 4 or greater. This criterion emphasizes the limiting effect of noise on the resolving capability of imaging systems. Unlike the Rayleigh criterion, the Rose criterion incorporates not only the overlap of intensity distributions but also the statistical characteristics of noise.

As schematically illustrated in Figure 7c, where the blue line represents the SEM linescan profile of two adjacent nanoparticles and the red line depicts the noise‐free profile, the signal intensity *S* is given as the depth of the central dip, and the noise intensity *N* corresponds to the standard deviation of the noise distribution. Here, differing from the Rayleigh criterion, the response function of the simulated imaging system contains additional blurring factors made of vibration and noise, i.e., $F=\mathrm{PSF}\cdot F_{v}\cdot F_{N}$. Therefore, $I=F_{N}\left\{F_{v}\left[I_{\mathrm{MC}}⊗\mathrm{PSF}\right]\right\}$. Noise is added to the image by the operations given in Equations (13) and (14) until the lower boundary of SNR=S/N≥4 is satisfied, and below which the two particles are considered unresolvable. To be resolvable the interparticle separation must be expanded. In case SNR is still under 4 when further increasing separation, the image noise is at such a great level that the image features are indistinguishable; therefore, the resolution determination becomes meaningless.

In the analysis of an actual SEM image the noise level *N* is calculated by the methodology outlined in ISO/TS 24597 [10] for assessing the contrast‐to‐noise ratio, defined as the standard deviation of the difference between the noisy image *I*(*i*, *j*) and the image obtained after applying a 3×3 median filter three times *I*_med_(*i*,*j*):

(18) $$N=\sqrt{\frac{1}{i_{\max}\times j_{\max}}}\sum_{j=1}^{j_{\max}}\sum_{i=1}^{i_{\max}}{\left[I_{\mathrm{med}}\left(i,j\right)-I\left(i,j\right)\right]}^{2}$$

The appropriate SEM image resolution should be taken as the larger value among those evaluated by the Rayleigh criterion and the Rose criterion (see Section S1.3):

(19) $$R=\max\left\{R_{\mathrm{Rayleigh}},R_{\mathrm{Rose}}\right\}$$

Furthermore, the resolution measurement is intimately linked to the pixel size. According to the Nyquist‐Shannon sampling theorem, the sampling frequency must be at least twice the maximum frequency present in the signal. Therefore, the measured resolution must be at least double the pixel size, which needs to be considered in practice of resolution determination.

#### 4.3.4 Comparison between Sharpness and Resolution

**FIGURE 8:**
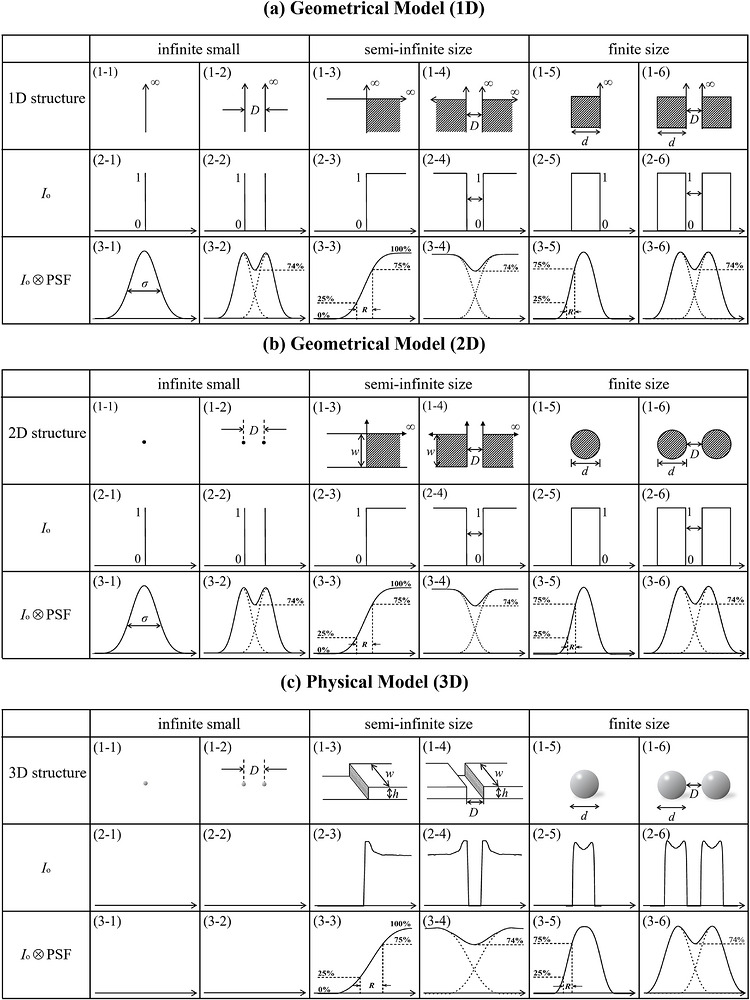
Schematic comparison on the concepts of sharpness and resolution, where the “geometrical model” represents the model adopted in ISO/TS 24597 [10] for the sharpness evaluation and the “physical model” is the one employed in this work for the resolution measurement. (a) Geometrical model (1D); (b) Geometrical model (2D); (c) Physical model (3D).

To further elucidate the concepts and definitions of sharpness and resolution and to clarify their differences, we illustrate in Figure 8 the modeled sample structure, ideal image *I*_o_(*x*) and the blurred image *I*_o_⊗PSF as considered by the geometrical model adopted in ISO/TS 24597 [10] for the sharpness evaluation, and the physical model in this work for resolution evaluation. The definitions of sharpness and the corresponding resolution in each case are shown. In the geometrical model, the sample is assumed to be the 1D semi‐infinite structures, while the physical model deals with 3D structures of finite sizes. Sharpness definition (as $\sqrt{2}\sigma \left(=R_{24\%-76\%}\right)$ in ISO/TS 24597 [10]), Figure 8a(3‐3), is given for the 1D semi‐infinite structure (Figure 8a(1‐3)), whereas in the discussion (E.4 of [10]) of corresponding resolution the referred structure is of the 2D finite size (Figure 8b(1‐6)) because it was argued that the Rayleigh criterion (Figure 8b(3‐2)) for the infinite small structure (Figure 8b(1‐2)) is not suitable. But this correspondence is obviously inappropriate, and the reasonable definition of the corresponding resolution for the modeled 1D semi‐infinite structure should be that as shown in Figure 8a(3‐4), which is evaluated as R = 0.66*σ* and significantly smaller than $\sqrt{2}\sigma$. Moreover, within the 2D geometrical model both the definitions of sharpness and resolution (Figure 8b(3‐5),(3‐6)) should be associated with the finite sized 2D structure (Figure 8b(1‐5),(1‐6), respectively). In Supplementary Methods the *R* − R conversion curves for the 1D and 2D geometrical models shown respectively in Figure S8a,b is compared with the Figure 3a or Figure S8c for the present physical model. Figure S8 then demonstrates the extent of approximation adopted in geometrical modeling in comparison with the present physical modeling.

It can be understood from the above discussion on the comparisons given in Figure 8 that the exact definitions of sharpness and resolution should be based on the physical model (Figure 8c(3‐5),c(3‐6)), incorporating all the physical factors about the sample (3D structure, size and chemical property), the beam (energy and width) and the detector (signal component) conditions. Therefore, not only sharpness *R* = *R*(*σ*, *d*, *E*…) depends on these factors but also R = R(*σ*, *d*, *E*…) does. A further in‐depth insight on the *R* − R relationship can be gained from the Shannon's rate‐distortion theory [66]. An edge contour shape contains full information about all the physical factors involved in beam‐sample interaction (sample chemical property, 3D morphology and size, beam energy and width, and signal type). In the model‐based library method a Monte Carlo method is used to generate simulated linescan profiles depending on all the modeled physical parameters [67]. This process establishes a mapping between profiles and physical parameters, and the 3D characteristic dimensional sizes can be measured from a modeled library by matching the experimental linescans with the simulated profiles. The present *R* − R method resembles the model‐based library method through a similar mapping process. In fact, one does not necessarily need the sharpness value to derive the resolution value. However, because a single value of R is insensitive to the details of linescan shape and an index, sharpness *R*, can be used to characterize the most important feature of linescan for the determination of R. This achieves information compression via a quotient space with allowable information distortion, i.e., not storing the full original information of linescan, but instead a *R*.

#### 4.3.5 Sharpness‐Resolution Conversion

##### 4.3.5.1 Implementation

**FIGURE 9:**
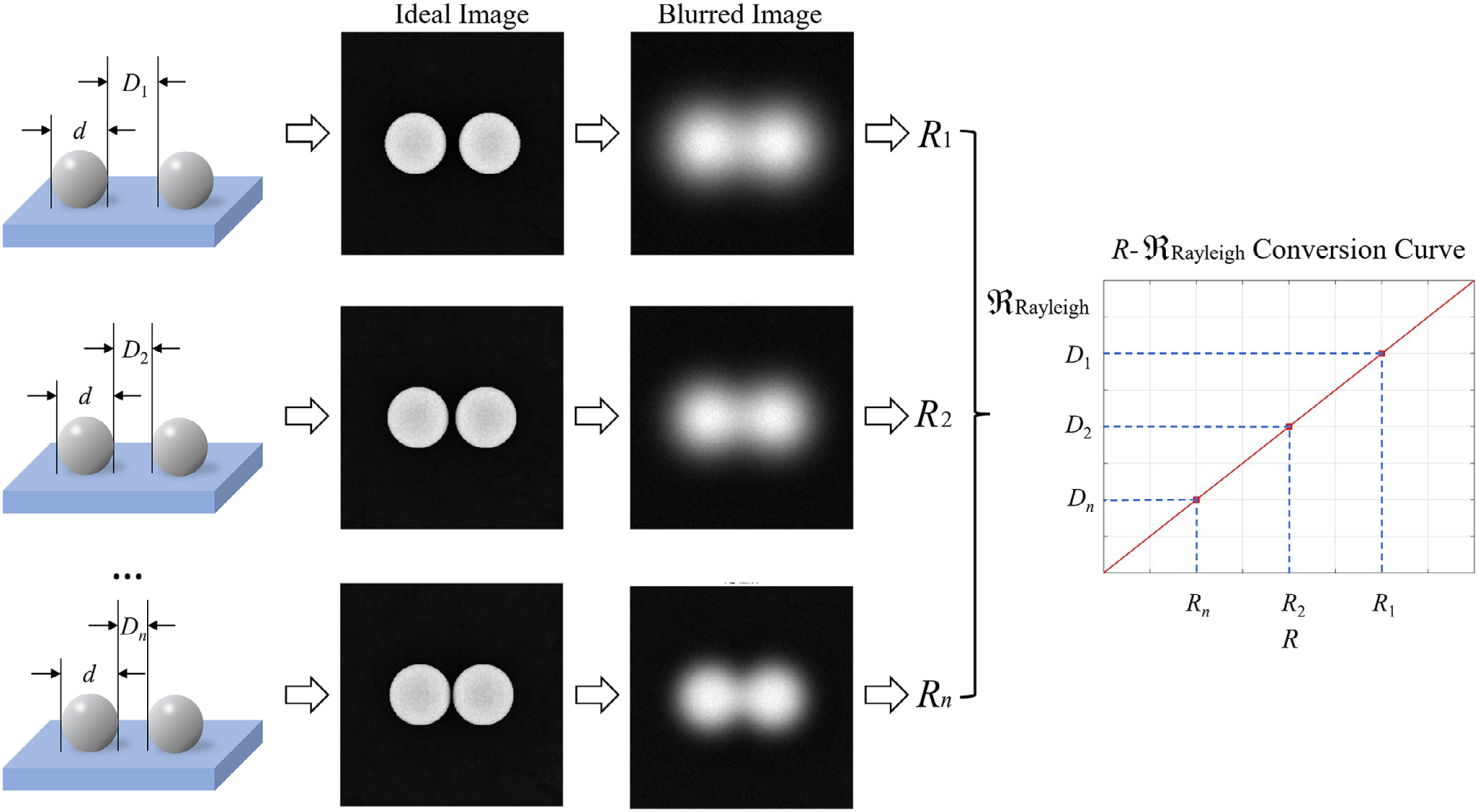
Schematic illustration of the method for building the sharpness‐resolution conversion curve, where the ideal image is *I*_o_ = *I*_MC_ and the blurred image is *I*_MC_⊗PSF.

Systematic Monte Carlo simulations are necessary to generate a series of SEM images $I_{o}\left(x,y\right)=I_{\mathrm{MC}}\left(x,y\right)$ of two identical gold nanoparticles on a carbon substrate plane with a specified interparticle spacing *D*. Critical conditions for the Rayleigh criterion are then achieved by modulating response function PSF(*x*, *y*) = *g*(*x*, *y*), i.e., changing the experimental parameters of focus and astigmatism, in Equation (9) for image blurring such that further blur will cause the particles to become unresolvable. According to the ISO terminology definition of SEM image resolution, the Rayleigh resolution is just the corresponding spacing *D* at this condition, $R_{\mathrm{Rayleigh}}$ = *D*. The sharpness *R* of the $I_{o}\left(x,y\right)⊗\mathrm{PSF}\left(x,y\right)$ image was then computed by using the improved DR algorithm as detailed in S1.1 of Supporting Information (together with the modified FT method and the present sharpness definition) to evaluate the gradient of an extracted edge profile and by taking average over all edge profiles of all particles in the image, establishing a quantitative mapping between image sharpness *R* and resolution $R_{\mathrm{Rayleigh}}$. A (*R*, $R_{\mathrm{Rayleigh}}$)‐data point of a 2D plot is then obtained. By varying the interparticle spacing *D* and repeating the above procedure, a series of sharpness‐resolution data points are produced to establish the sharpness‐resolution conversion curve (*R* − $R_{\mathrm{Rayleigh}}$ curve). The entire process is schematically shown in Figure 9.

In a Monte Carlo simulation of SEM image, a sufficient number of image pixels is necessary (at least 400×400 here); for each pixel position, usually ∼10^3^ or more incident electron trajectories are tracked down to the energy of inner potential. Because the curve depends on the beam energy and particle size (and additionally the particle shape, particularly for large particle sizes where the particle shape substantially deviates from the spherical‐like in a practical resolution sample, as discussed in Section S3 on the uncertainty of the conversion curve) a comprehensive simulation is indispensable. Similarly, by adjusting other response function parameters for vibration and noise so that the blurred noisy SEM image $F_{N}\left\{F_{v}\left[I_{o}\left(x,y\right)⊗\mathrm{PSF}\left(x,y\right)\right]\right\}$ just meets the Rose criterion, one *R* − $R_{\mathrm{Rose}}$ curve will be generated, where the corresponding spacing *D* determines the Rose resolution, $R_{\mathrm{Rose}}$ = *D*. The Rose and Rayleigh criteria yield analogous results in the resolution conversion process; however, the Rose criterion incorporates the effect of image noise by adjusting the critical dip‐to‐peak ratio based on the prevailing noise intensity. Then the noise level *N* acts as another dimensional variable in sharpness‐resolution conversion, and “*R* − $R_{\mathrm{Rose}}$ curve” is actually a surface other than a curve. In addition, an accurate noise assessment also requires consideration of image contrast. To ensure the validity of Rose criterion‐based resolution conversion, the simulated $F_{N}\left\{F_{v}\left[I_{o}\left(x,y\right)⊗\mathrm{PSF}\left(x,y\right)\right]\right\}$ SEM images must match the required contrast for the experimental one [10]. This can be achieved through an appropriate contrast rescaling during the simulation, where the Michelson contrast [68] is used to quantify the contrast of SEM images, which is defined as:

(20) $$C=\frac{I_{\max}-I_{\min}}{I_{\max}+I_{\min}}$$

where *I_max_* and *I_min_* represent the maximum and minimum gray values in the simulated SEM image, respectively.

##### 4.3.5.2 Application

In essence, the *R* − $R_{\mathrm{Rayleigh}}$ conversion curve establishes an exact one‐to‐one correspondence between the sharpness *R* in an experimental SEM image and resolution $R_{\mathrm{Rayleigh}}$ value for a simulated SEM image to meet the Rayleigh criterion under the ISO terminology definition of SEM image resolution. In practice of image sharpness evaluation for an experimental SEM image, a *R*‐value for a particular edge profile of a particle of diameter *d* is evaluated via the defined gradient *R*_25*%* − 75*%*_, which is then mapped into the $R_{\mathrm{Rayleigh}}$‐value from the *R* − $R_{\mathrm{Rayleigh}}$ conversion curve. Therefore, the conversion curve is functioned like a “calibration curve”. The image resolution is obtained from statistical averaging of all the $R_{\mathrm{Rayleigh}}$‐values calculated for all the successfully extracted particle edge profiles for those particles in a specified range of size *d*. The image sharpness of the SEM is also obtained as the average of the local sharpness values for the sake of comparison. Similarly, the $R_{\mathrm{Rose}}$ value can also be derived from the *R* − $R_{\mathrm{Rose}}$ conversion curve once the noise level *N* of the whole image is evaluated. The test results with several different SEM instruments at different magnifications are reported in Table S1. For the two instruments, the obtained R‐values at two higher magnifications are nearly equal, while the *R*_DR_‐values differ somewhat, and the *R*_DR_ (ISO)‐values differ a lot. Particularly, *R*_DR_ (ISO) tends to be smaller at higher magnifications but R is rather stable. This fact indicates that the resolution R is a much better metric than the sharpness *R* for representing instrumental capability, i.e., the resolving power.

## Author Contributions

**Tongfang Yang**: conceptualization, methodology, software, computation, formal analysis, writing – original draft, visualization. **Yanbo Zou**: investigation, formal analysis, funding acquisition. **Zejun Ding**: conceptualization, methodology, software, formal analysis, writing – review & editing, supervision, funding acquisition, project administration.

## Conflicts of Interest

The authors declare no conflicts of interest.

## Supporting information




**Supporting File**: advs75710‐sup‐0001‐SuppMat.pdf.

## Data Availability

The data that support the findings of this study are available from the corresponding author upon reasonable request.
