# Supplementary material for: On Determination Method for Resolution of Secondary Electron Images in Scanning Electron Microscopy
Source: Adv Sci (Weinh). 2026 May 25:e19630. Online ahead of print. doi: 10.1002/advs.202519630 (PMC13336055; doi:10.1002/advs.202519630)
Supplement: Supplementary file 1 — Supporting File: advs75710‐sup‐0001‐SuppMat.pdf. [file ADVS-9999-e19630-s001.pdf]

Supporting Information for On Determination Method for Resolution of Secondary Electron Images in Scanning Electron Microscopy

T. F. Yang[1], Y. B. Zou[2#] and Z. J. Ding[1*]

[1]*Hefei National Research Center for Physical Sciences at the Microscale and Department of Physics, University of Science and Technology of China, Hefei, Anhui 230026, P.R. China*

[2]*School of Physics & Electronic Engineering, Xinjiang Normal University, Urumqi, Xinjiang 830054, P.R. China*

[#]Corresponding author: zouyb@xjnu.edu.cn

[*]Corresponding author: zjding@ustc.edu.cn

This PDF file includes:

S1. Supplementary Methods

S1.1 Improved DR Method

S1.2 Sharpness

S1.3 Resolution

S2. Sensitivity Analysis

S3. Uncertainty Analysis

S4. Detector Type

S5. Supplementary Results

S1. Supplementary Methods

This work concentrates on the resolution of secondary electron (SE) images in scanning electron microscopy (SEM) and the related sharpness-resolution conversion method. We describe below the details of the involved concepts and methods.

S1.1 Improved DR Method

This section systematically introduces the derivative method (DR) for evaluating sharpness of SEM images. The core idea of the DR method is to fit the gray value transition across an edge of particle with an error function and to extract a sharpness-related quantity from the fitting parameters. If the system point spread function (PSF) is assumed to follow a Gaussian distribution, the corresponding edge spread can be approximated by an error function. Specifically, the edge profiles along the normal direction to the boundary of a given particle are selected and fitted individually with an error function. The image sharpness of that particle is then obtained by averaging the fitting results over all selected profiles.

The DR method used in this work is developed by improving and extending the original DR method given in the international standard, ISO/TS 24597 [1]. While retaining its mainstream, the algorithm has been optimized in terms of implementation details, robustness, and applicability. The main modifications are as follows: (1) A threshold determination scheme combining two methods, original one and Otsu method, is adopted in the image binarization stage to improve robustness; (2) The edge-labeling algorithm is revised as the original one may produce internal holes or discontinuities in the labeling results; (3) Additional procedure for particle labeling and the corresponding particle-size statistics are incorporated for the application of sharpness-resolution conversion curve which is particle-size dependent; (4) The Sobel operator is used to calculate image gradients; (5) The original DR method uses a fixed edge curve length, which is not universally applicable to the practical SEM images in different magnifications and may cause large uncertainty of curve fitting. To improve its adaptability, the present algorithm dynamically determines the optimal edge curve length for the DR analysis of a specific image based on a preliminary test of image sharpness by the Fourier

transform (FT) algorithm defined in ISO/TS 24597 [1]. This step imposes more reasonable edge-profile screening criteria and improves robustness.

On this basis, the DR calculation procedure adopted in this work can be divided into four main steps: image preprocessing and binarization, particle and edge identification, extraction and fitting of edge profiles, and final calculation of image sharpness from the fitted parameters. These steps are described below in sequence.

First, the original SEM image is preprocessed to suppress random noise and improve the stability of edge identification. In this work, median filtering is applied to remove isolated noise points, and the image is then binarized on the basis of its grayscale histogram so as to distinguish particle regions from the background. When the grayscale histogram exhibits a clear bimodal distribution, a histogram-peak-based thresholding method is adopted. When the bimodal feature is not sufficiently pronounced and the image quality is relatively poor, the Otsu method is used instead to determine the binarization threshold. After binarization, appropriate morphological processing is further applied to fill small holes inside particles and to reduce the influence of edge burrs on the subsequent analysis.

Individual particles in the image are identified by connected-component labeling, and their boundaries are then extracted. Incomplete particles located near the image boundary, as well as excessively small particles, are excluded from the subsequent analysis to correctly determine the particle size and avoid unstable contributions to the sharpness statistics. At the same time, the equivalent size of each particle is recorded for the subsequent conversion from sharpness to resolution. After this treatment, a set of reliable particle-edge points is obtained as the input for the DR sharpness calculation.

For each valid edge point, the local grayscale gradient is first calculated by using the Sobel operator to determine the normal direction of the edge. An intensity profile is then extracted along this normal direction. Because local noise, neighboring particles, or edge irregularities in a realistic SEM image may distort the extracted profile, a screening procedure is applied to discard profiles with insufficient contrast, anomalous directionality, or obvious deviation from

a monotonic edge transition. Only those profiles that can reliably represent the true edge broadening are retained for fitting.

As shown in Figure S1, each retained edge profile is fitted with the error function,

$$f_j(x) = b_j + h_j\left[\frac{1}{2} + \frac{1}{2}\mathrm{erf}\left(\frac{x - m_j}{\sigma_j\sqrt{2}}\right)\right], \tag{S1}$$

where b_j, h_j, m_j and σ_j are fitting parameters for the *j*th profile. The parameter σ_j characterizes the degree of edge broadening. For each candidate edge point, eight different edge-profile lengths are used for fitting, and the corresponding root-mean-square errors (RMSEs) are calculated as

$$\mathrm{RMSE} = \sqrt{\frac{1}{4M+1}\sum_{x=-2M}^{2M}\left[P_j(x/2) - f_j(x/2)\right]^2}\,. \tag{S2}$$

where P_j is the gray value of the *j*th profile. The number of data points is taken as $4M+1 = \mathrm{int}\{(4, 6, 8, 11, 14, 18, 23, 29)R_{\mathrm{FT}}\}$, where R_{FT} is the initial estimation of the image sharpness by the FT method. The edge profile giving the minimum RMSE is selected to deduce the final fitting result for the profile.

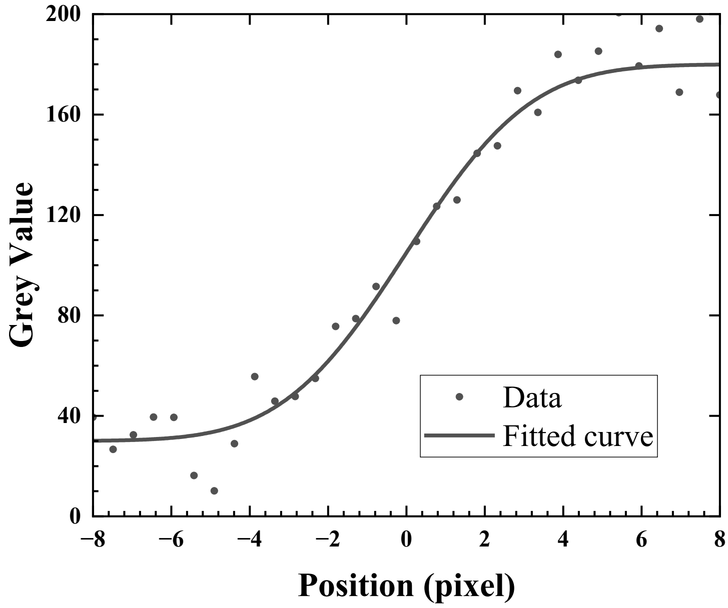

Figure S1 Schematic illustration of fitting an edge profile in an SEM image with an error function.

Here, the DR sharpness is defined by the 25%–75% criterion between the maximum and minimum of the fitted edge profile, i.e., the lateral distance over which the fitted intensity rises from 25% to 75% of its full amplitude as shown in Figure S2, although the final converted resolution value does not depend critically on the selected threshold values in sharpness definition. To compare with the sharpness definition specified in ISO/TS 24597 [1] with the σ of the fitted error function, the relationship is given by,

$$R_{\mathrm{DR}} = R_{25\%-75\%} = 1.349\sigma. \tag{S3}$$

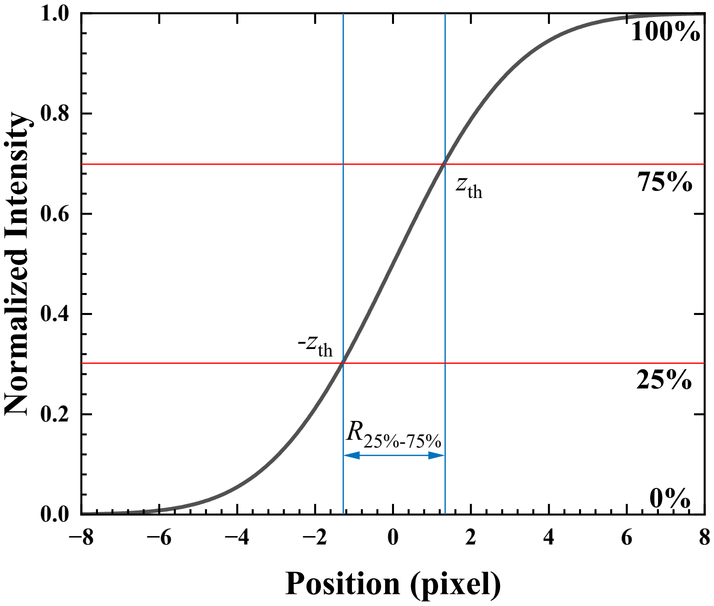

Figure S2 Schematic illustration of the 25%–75% sharpness definition for a particle edge profile.

S1.2 Sharpness

This section compares the sharpness concepts in the geometrical model and physical model. Figure S3 shows the evaluated sharpness ($R_{25\%-75\%} \equiv R$) of edge profiles from the convoluted image, $I(x,y) = I_{\mathrm{o}}(x,y) \otimes \mathrm{PSF}(x,y)$, in the case of 2D finite size for the geometrical model, where $I_{\mathrm{o}}(x,y) = W(x,y)\left\{=1, \sqrt{x^2+y^2} < d; = 0, \text{otherwise}\right\}$ is a 2D circular window function as a representation of the ideal particle image of diameter d by the geometrical model, PSF is a 2D Gaussian function, $G(x,y;\sigma) = \exp\left[-\left(x^2+y^2\right)/2\sigma^2\right]/2\pi\sigma^2$, i.e. Eq. (8)

for ($s=1$, $\varphi_s=0$). The result shows that R is nonlinear with the focus factor σ, and can be approximated as,

$$R=\begin{cases}1.35\sigma, & if\ d>5\sigma;\\ 0.11d+0.82\sigma, & if\ d<5\sigma.\end{cases} \quad \text{(S4)}$$

For large particles, $R_{25\%-75\%} \sim 1.35\sigma$ approaches the relationship for the 1D semi-infinite geometrical model. Considering the 3D finite size effect and physical factors, the approximations made for $R_{24\%-76\%}=\sqrt{2}\sigma$ will be less valid for small particles of $d<5\sigma$.

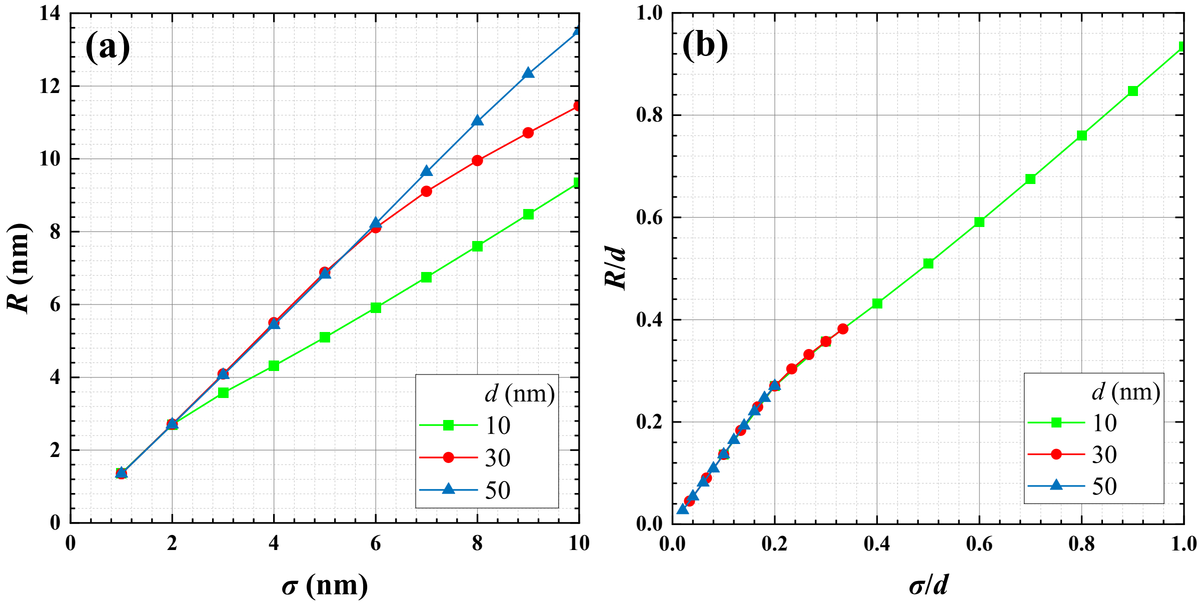

Figure S3 (a) The sharpness $R_{25\%-75\%}$ as a function of beam focus factor σ and circular particle diameter d evaluated from a 2D circular finite structure and 2D PSF Gaussian function by a geometrical model shown in Fig. 8(1-6); (b) reduced sharpness R/d as a function of reduced focus factor σ/d.

In practice, different sharpness metrics are adopted for the measure of profile gradient by different researchers and manufacturers. The commonly used one is the $R_{25\%-75\%}$ ($\sim 1.35\sigma$ for 1D geometrical model, and the formula (S4) for 2D geometrical model). Alternative sharpness metrics include $R_{15\%-85\%}(\sim 2.07\sigma)$, $R_{16\%-84\%}(\sim 1.99\sigma)$ adopted by JEOL Inc. [2], $R_{24\%-76\%}(\sim \sqrt{2}\sigma)$ specified in ISO/TS 24597 [1], and $R_{35\%-65\%}(\sim 0.77\sigma)$.

It is worth mentioning that with this DR method the edge intensity profiles in the gap region between two particles are practically excluded in the present calculation of sharpness if the gap under the Rayleigh criterion is very narrow (below three pixels), because the edge curve length is too short to be useful; then in fact only those edges well away from the gap region enter the evaluation.

To demonstrate the approximation made in the geometrical model, Fig. S4 illustrates example calculations of the convoluted image intensity $I(x)$ for a 1D finite size structure with assumptions of $I_{\mathrm{o}}(x)=\Theta(d/2+x)\Theta(d/2-x)$ and $\mathrm{PSF}(x)=G(x;\sigma)$, where d is particle diameter. The top panel (Figs. S4(a) & S4(d)) shows the linescan profiles $I_{\mathrm{o}}(x)$ over the range of two edges of a particle in different sizes, $d=10$ nm and $d=100$ nm. The middle panel (Figs. S4(b) & S4(e)) and the bottom panel (Figs. S4(c) & S4(f)) display the corresponding $I(x)$ profiles by convolution with Gaussian functions of smaller ($\sigma=1$ nm) and larger ($\sigma=5$ nm) beam focuses, respectively. The evaluated edge sharpness is $R=1.35\sigma$, consistent with the definition, if the particle size is much greater than the beam diameter ($d \gg \sigma$), as shown by Figs. S4(b) & S4(e). But, when the beam diameter becomes larger the finite particle size will begin to show the effect (Fig. S4(c)) and the sharpness is thus smaller than the simple estimation of 1.35σ, i.e. $R<1.35\sigma$, and larger the σ/d smaller the R. Therefore, it can be understood that even under the simplification of geometrical model by ignoring physical factors, the sharpness evaluation still should involve the size factor, but not as assumed in the DR algorithm in ISO/TS 24597 [1]. Moreover, in reality, due to physical factors the ideal intensity distribution of a particle can no longer be the simple step function like, and instead exhibits a certain profile shape (e.g. the edge bloom, known as the edge effect). This behavior can be seen from a Monte Carlo simulated linescan $I_{\mathrm{o}}(x)=I_{\mathrm{MC}}(x,y=0)$ illustrated in Figs. S5(a) & S5(d) for a 3D particle. When $I_{\mathrm{o}}(x,y)=I_{\mathrm{MC}}(x,y)$ is convolved with a 2D $\mathrm{PSF}(x,y)=G(x;\sigma)G(y;\sigma)$ to derive linescan $I(x,y=0)$, a narrower beam width ($\sigma=1$ nm) produces edge sharpness values $R>1.35\sigma$ for both $d=10$ nm and

$d = 100$ nm, shown in Figs. S5(b) & S5(e), respectively. Conversely, a larger focus parameter or a wider beam size ($\sigma = 5$ nm) produces edge sharpness values $R < 1.35\sigma$ (Figs. S5(c) & S5(f)). It is obvious that the sharpness R relates to σ nonlinearly in the physical model, exemplifying its close relationship with physical factors, $R = R(\sigma, d, E...)$, and emphasizing the necessity of considering these factors in the sharpness and resolution evaluation. By the 2D geometrical model, at least the factor of diameter should be concerned as in Eq. (S4) instead of using the simple relationship $R_{24\%-76\%} = \sqrt{2}\sigma$.

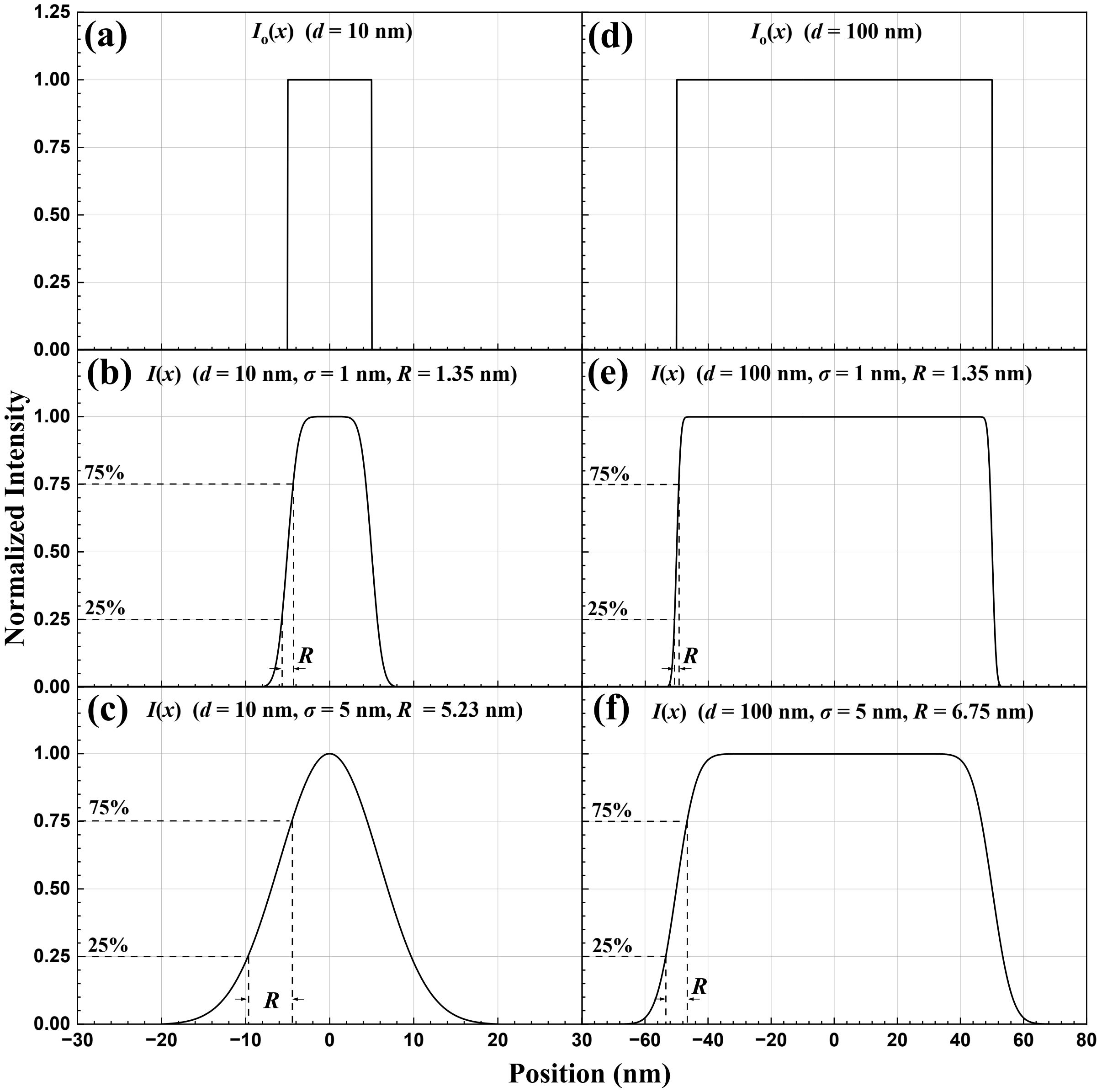

Figure S4 Image intensity profile $I(x)$ for a finite sized particle in 1D geometrical model shown in Fig. 8(1-5) as convolution of the assumed ideal intensity

$I_o(x) = \Theta(d/2 + x)\Theta(d/2 - x)$ with a Gaussian PSF $\mathrm{PSF}(x) = G(x;\sigma)$, where d is particle diameter, σ the standard deviation of the Gaussian function and R the evaluated sharpness.

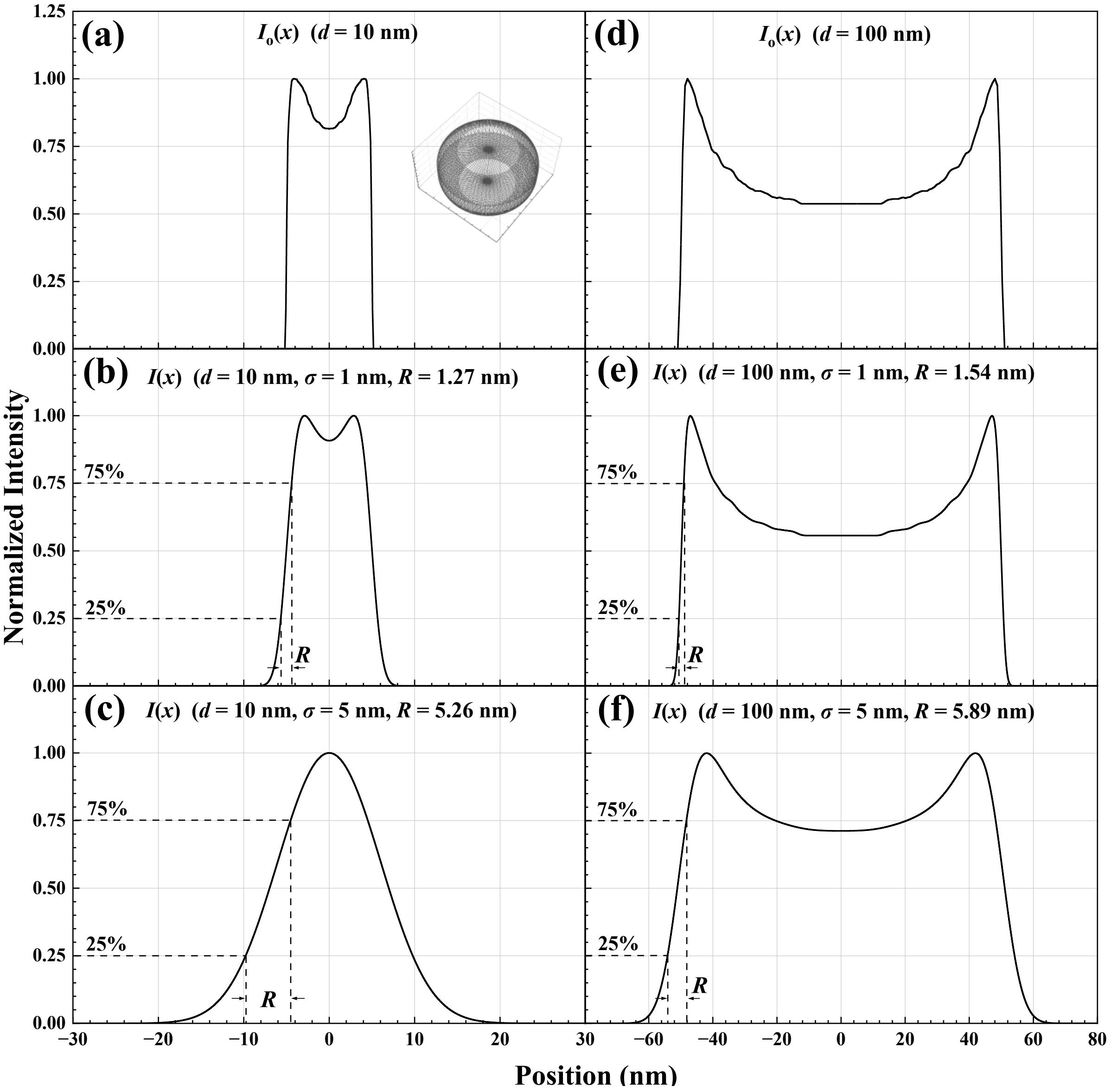

Figure S5 Image intensity profile $I(x, y=0) = I_o(x, y) \otimes \mathrm{PSF}(x, y)\big|_{y=0}$ for a 3D particle as convolution of the Monte Carlo simulated ideal intensity $I_o = I_{MC}$, for a 1 keV beam, with a 2D Gaussian PSF, where d is particle diameter, σ is the standard deviation of the Gaussian function or the beam diameter and $R_{25\%-75\%}$ is the sharpness. The particle morphology is described in the inset of (a).

S1.3 Resolution

This section compares the resolution concepts under the Rayleigh and the Rose criteria. A comprehensive assessment was conducted in sharpness-resolution conversion by considering

both the Rayleigh and the Rose criteria to evaluate the combined influences of response function on SEM image resolution. Figure S6 presents a comparison of the resolution ($\Re$) by the two criteria under varying noise levels, *N*. Figs. S6(a)-S6(d) display grayscale intensity distributions based on the Rayleigh criterion, while Figs. S6(e)-S6(h) show the corresponding results based on the Rose criterion (SNR of at least 4 is needed to be able to distinguish image features, as illustrated in Fig. 7). The noise level *N*, quantified by Eq. (17), increases progressively from the top panel to the bottom panel in Fig. S6. When the noise level is quite low (Figs. S6(a) & S6(e)), the resolution is primarily limited by the optical system (i.e. the beam diameter σ) and the Rayleigh criterion ($\Re_{\mathrm{Rayleigh}}$) provides the more appropriate estimate of resolution. At moderate noise levels (Figs. S6(b) & S6(f)), the resolutions obtained from both criteria are equal. As the noise increases further (Figs. S6(c) & S6(g)), the resolution becomes predominantly limited by the noise in signal detection, and the Rose criterion ($\Re_{\mathrm{Rose}}$) yields a more suitable estimation. Under an extreme high noise condition (Figs. S6(d) and S6(h)) of SNR < 4, even the Rose criterion may not be reached, and both the criteria are not applicable. In each figure the gold particle configuration whose separation satisfies the criterion is shown, where the Rayleigh criterion yields the same resolution value for all the noise levels while the Rose criterion derives the poorer resolution for the higher noise level. In summary, it is easy to understand from this comparison that the reasonable SEM image resolution should be taken as the greater one among those obtained from the Rayleigh and Rose criteria.

Figure S7 provides a plot of Rayleigh resolution $\Re_{\mathrm{Rayleigh}}$ for a pair of 2D circular spots in a finite size corresponding to the respective sharpness $R_{25\%-75\%}$ shown in Figure S3. Similar to Eq. (S4), we have found that $\Re_{\mathrm{Rayleigh}}$ is nonlinear with the focus parameter σ, and can be approximated as,

$$\Re = \begin{cases} 0.4\sigma, & if\ d > 3.7\sigma; \\ -0.49d + 2.2\sigma, & if\ d < 3.7\sigma. \end{cases} \tag{S5}$$

Then both Eqs. (S4) & (S5) contain the finite size effect, which are a better approximation than the present sharpness definition of $\sqrt{2}\sigma$.

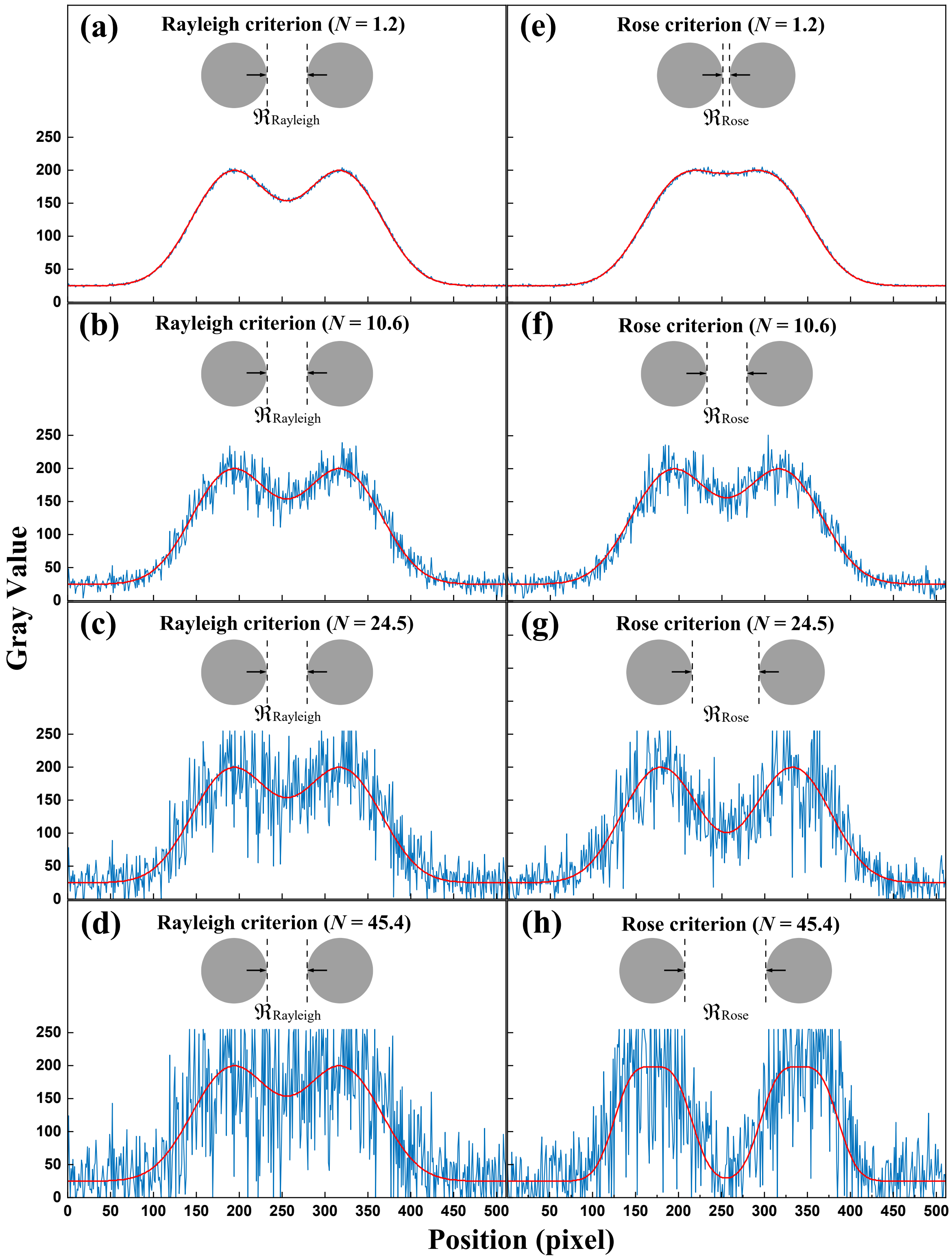

Figure S6 Comparison of grayscale linescan profiles under the Rayleigh criterion (left column) at $\sigma = 8.82$ nm and the Rose criterion (right column) at varied σ for different noise levels. The red line represents linescan $I_{\text{MC}}(x,y) \otimes \text{PSF}(x,y)\big|_{y=0}$ for a 15 keV electron beam

incident onto two separated gold particles ($d = 20$ nm), and blue line is $\mathbb{F}_N\{I_{MC} \otimes \text{PSF}\}$ by adding noises.

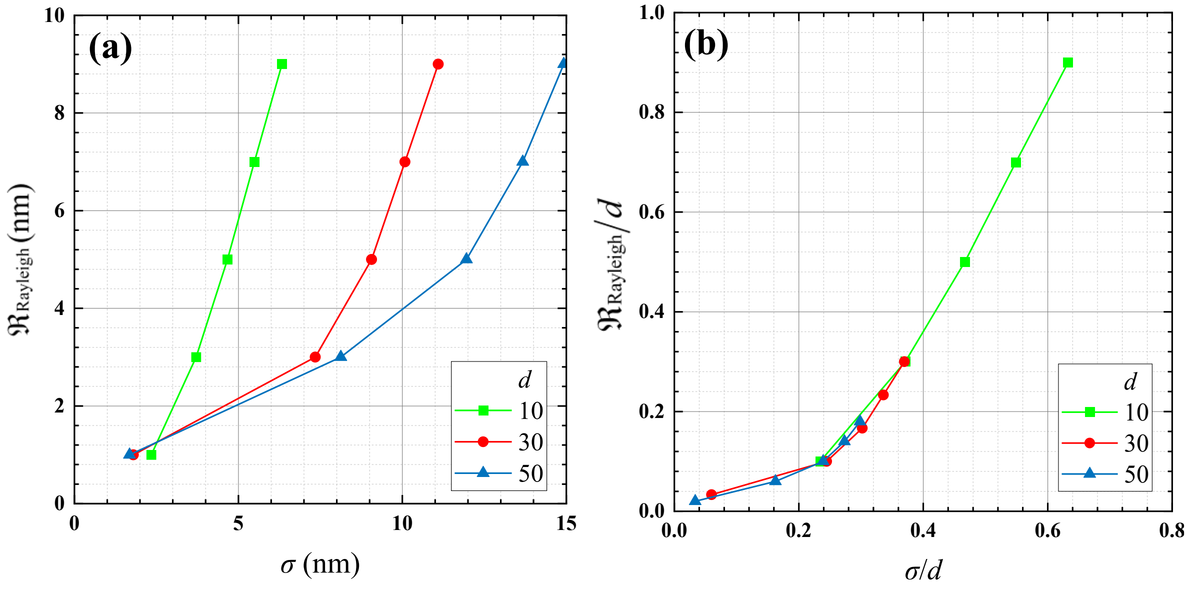

Figure S7 (a) The Rayleigh resolution $\Re_{\text{Rayleigh}}$ as a function of beam focus factor σ and circular particle diameter d evaluated from a 2D circular finite structure and 2D PSF Gaussian function by a geometrical model shown in Fig. 8(1-6); (b) reduced Rayleigh resolution $\Re_{\text{Rayleigh}}/d$ as a function of reduced focus factor σ/d.

Following the workflow described in section 4.3.5, the sharpness-resolution conversion curves have been generated for 1D & 2D geometrical models, as shown in Figs. S8(a) & S8(b), respectively. In Fig. 8 the corresponding conversion curves for the physical model shown in Fig. 3(a) are also presented in Fig. S8(c) for comparison. It is obvious that, for small particles, the geometrical-model derived conversion curves are indeed quite close to that of the physical model. However, as the particle size increases, the conversion curves of the physical model deviate significantly from those of the geometrical models. This difference arises mainly because the edge effect in an SEM image is insignificant for small particles, whereas for larger particles, neglecting edge effect leads to an error. The results further demonstrate that the physical factors involved in SEM imaging need to be adequately considered when evaluating resolution.

One may wonder whether the calculated resolution $\Re$ value changes with the definition of sharpness, being either the $R_{25\%-75\%}$ here or any others. Figure S9 explains that the choice of

the sharpness definition for the R–$\mathfrak{R}$ conversion curve does not affect the final resolution outcome (once the thresholds, α and $1-\alpha$, are well separated), where the sharpness serves as an index for transmitting the information of image blur or the linescan shape in an SEM observation to Rayleigh resolution under the "Rayleigh's SEM". As aforementioned, the previous definition of sharpness [1] based on a geometrical model has assumed one dimensionality of structure and 1D PSF and omitted all physical factors. In comparison, the present physical model-based R–$\mathfrak{R}$ conversion curve method provides an improvement and perhaps the ideal solution for the problem of SEM resolution evaluation.

Because the calculated sharpness and resolution values now depend on the particle size (while the traditional idea about the resolution has been that it is unrelated to the particle size), it is necessary to quantify the diameters of individual particles identified in an SEM image linked to their respective edge profiles. This is done by calculating the pixel area occupied by a particle, A; the corresponding effective particle diameter d is estimated via $d=\sqrt{4A/\pi}$.

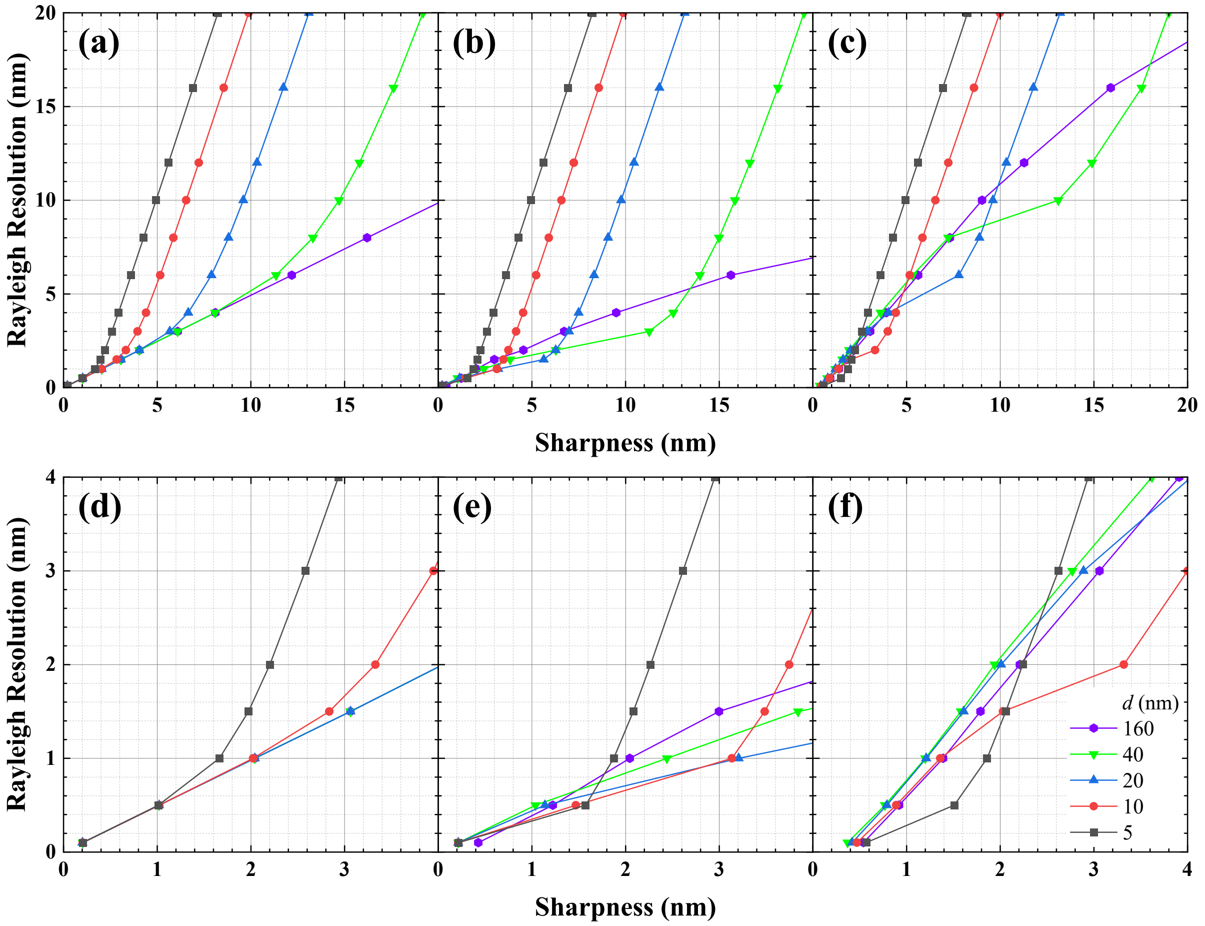

Figure S8 Comparison on the sharpness-resolution conversion curves between (a,d) 1D geometrical model, (b,e) 2D geometrical model, and (c,f) 3D physical model at a primary electron energy of 15 keV. The top and bottom panels present respectively the curves in wider and narrower ranges of sharpness and resolution values.

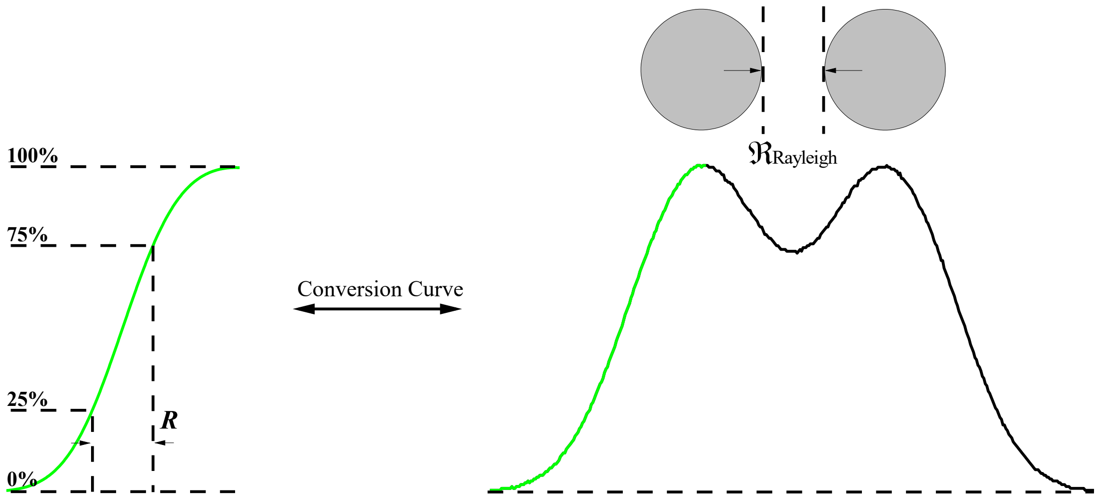

Figure S9 Schematic diagram for the correspondence between the sharpness *R* of an edge profile in a simulated/experimental SEM image and the resolution $\Re_{\text{Rayleigh}}$ of simulated image profile of two particles under the "Rayleigh's SEM", indicating that the resolution value does not depend on the choice of sharpness definition.

S2. Sensitivity Analysis

This part presents the simulated effects of different experimental factors on the evaluation results of sharpness and resolution. Here, the 3D gold-particle configuration (Figure S10(b)) constructed by Zhang et al. [3] by using a finite-element mesh modeling based on a realistic SEM image (Figure S10(a)) is adopted. Figure S10(c) shows the simulated ideal SEM image under perfect focus, without drift or vibration, at an incident electron energy of 15 keV. The sharpness evaluation methods used in this study, including the contrast-to-gradient method (CG), FT and DR (hereafter referred to as original DR) are all from an international standard [1]. In addition, the improved derivative method (improved DR) proposed in this work is also included for comparative testing.

Different degrees of defocus can be introduced through Eqs. (7)-(9). Simulated SEM images under different focusing parameters σ are generated from Figure S10(c), as shown in Figure S11, and the corresponding sharpness and resolution evaluation results are presented in Figure

S12. It can be seen that, all sharpness and resolution values increase with σ, exhibiting a trend consistent with physical intuition.

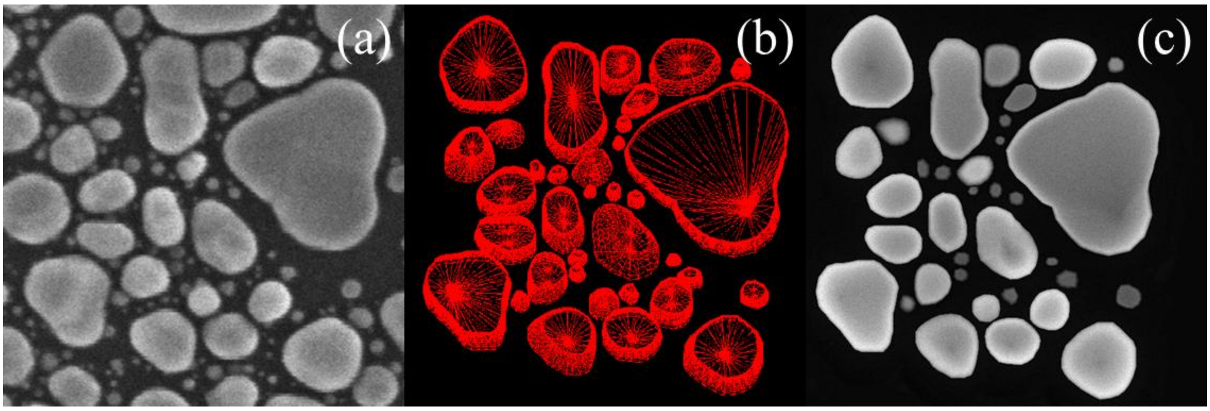

Figure S10 (a) Experimental SEM image of Au particles on a carbon substrate; (b) Au-particle configuration used for the Monte Carlo simulation; (c) Monte Carlo simulated SEM image (512×512 pixels) obtained under ideal instrumental conditions (perfect focus, without drift or vibration) with a 15 keV incident electron beam.

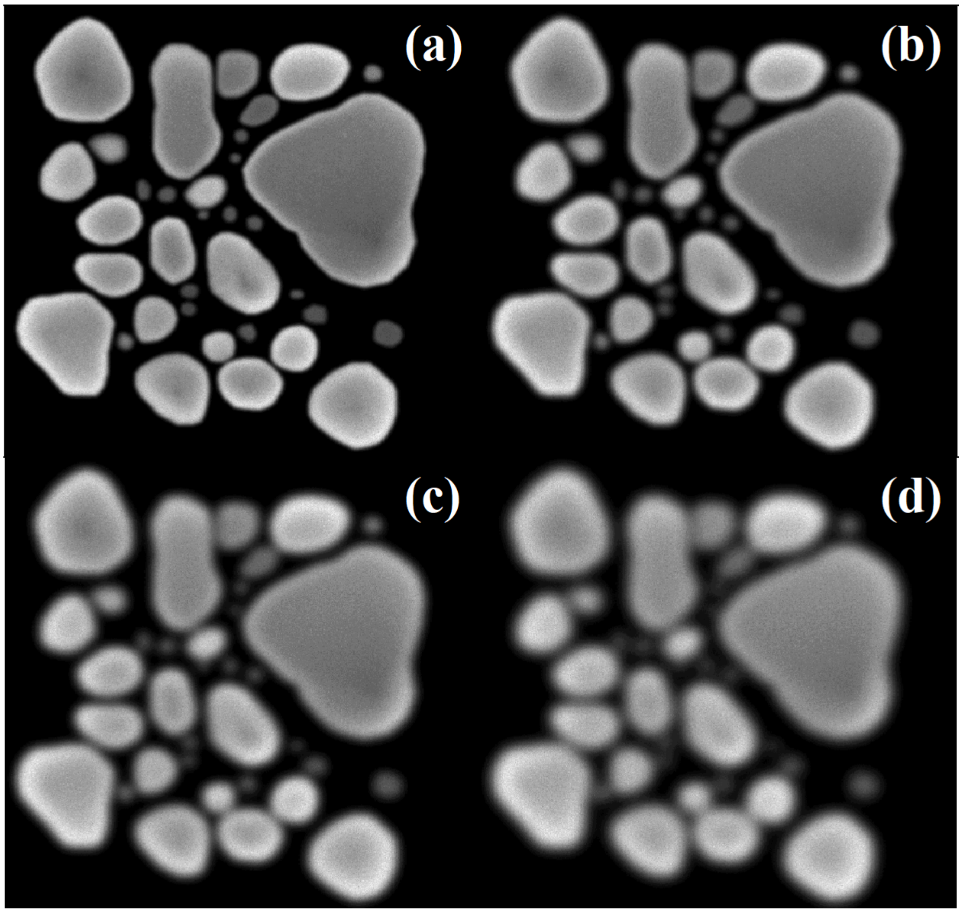

Figure S11 Simulated SEM images under different focusing parameters: (a) $\sigma = 1$ nm, (b) $\sigma = 2$ nm, (c) $\sigma = 3$ nm, and (d) $\sigma = 4$ nm.

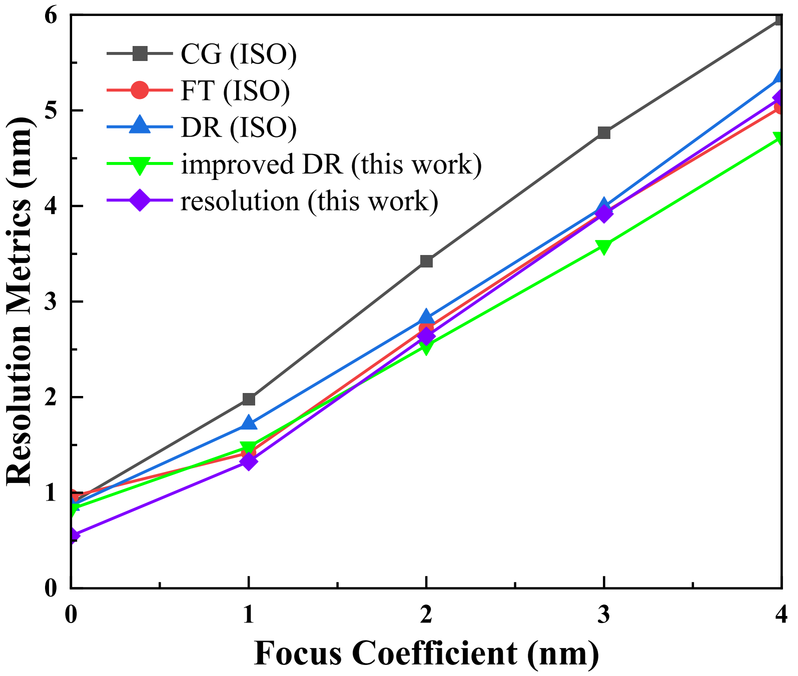

Figure S12 Sharpness and resolution of the processed SEM images in Figure S11 as functions of the focusing parameter σ.

According to Eqs. (7)-(9), simulated SEM images under different astigmatism parameters *s* are generated from Figure S10(c), as shown in Figure S13, and the corresponding sharpness and resolution evaluation results are presented in Figure S14. The extent to which the astigmatism parameter *s* deviates from 1 characterizes the degree of astigmatism. It can be seen that all sharpness and resolution values increase with increasing astigmatism, whereas the FT sharpness shows relatively low sensitivity to the degree of astigmatism.

According to Eqs. (10) and (11), simulated SEM images under different vibration amplitudes *A* are generated from Figure S10(c), as shown in Figure S15, and the corresponding sharpness and resolution evaluation results are presented in Figure S16. Among them, the sharpness values obtained by the CG and FT methods are insensitive to changes in vibration amplitude. In contrast, the sharpness values obtained by the original DR and the improved DR methods as well as the resolution values, all show the reasonable variation trend.

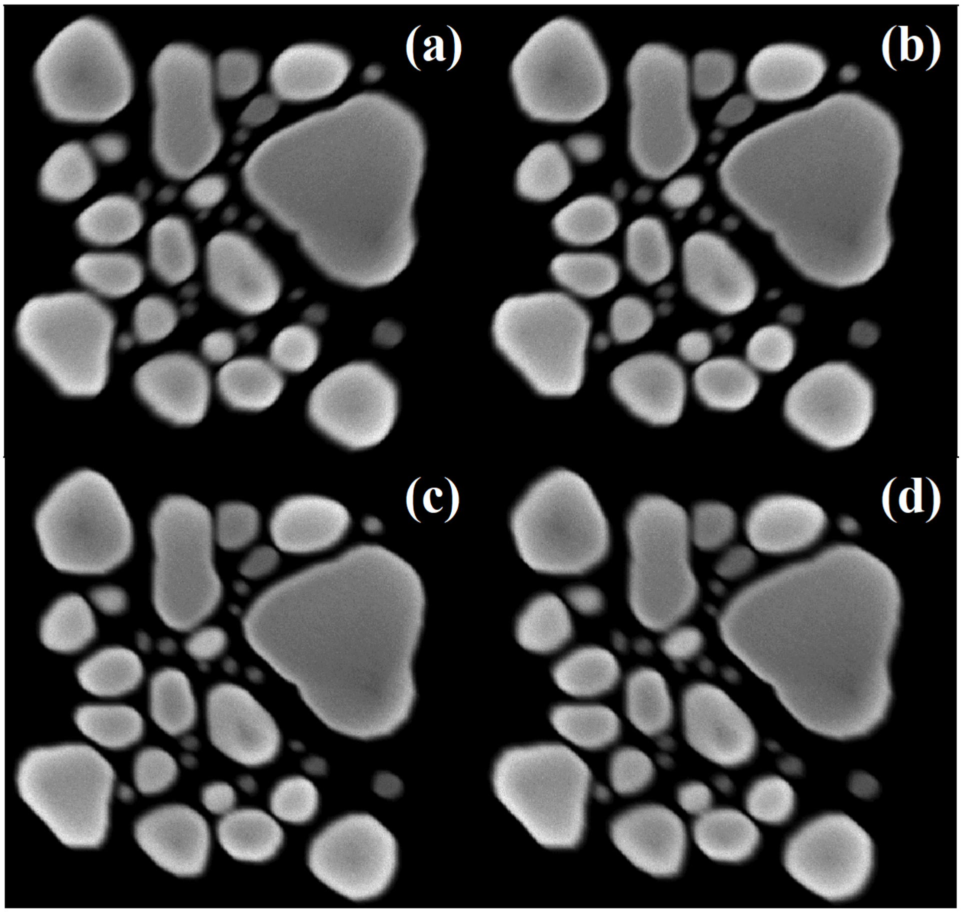

Figure S13 Simulated SEM images under different astigmatism parameters: (a) $s = 0.4$, (b) $s = 0.5$, (c) $s = 2.0$, and (d) $s = 2.5$. Here, $\varphi_s = 45^\circ$ φ and $\sigma = 1$ nm.

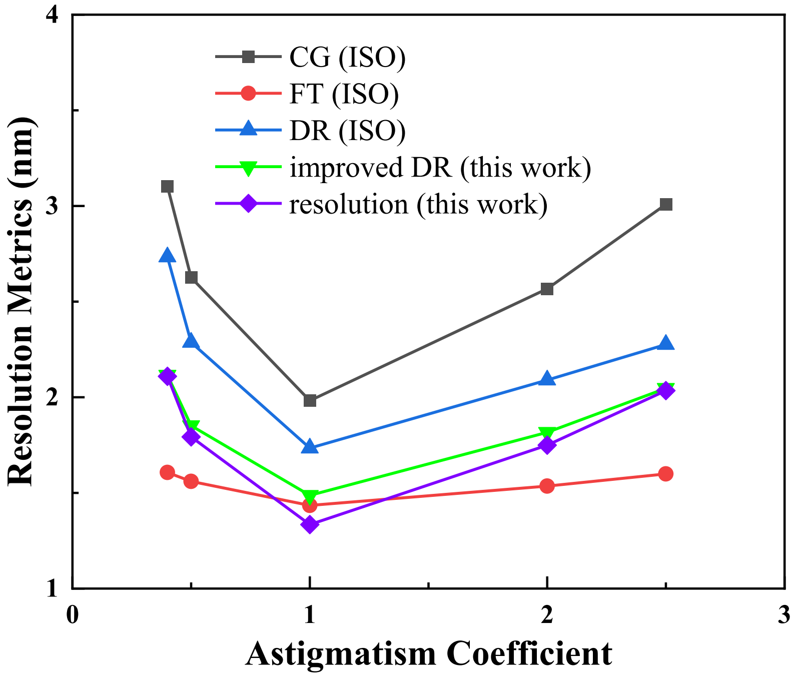

Figure S14 Sharpness and resolution of the processed SEM images in Figure S13 as functions of the astigmatism parameter *s*.

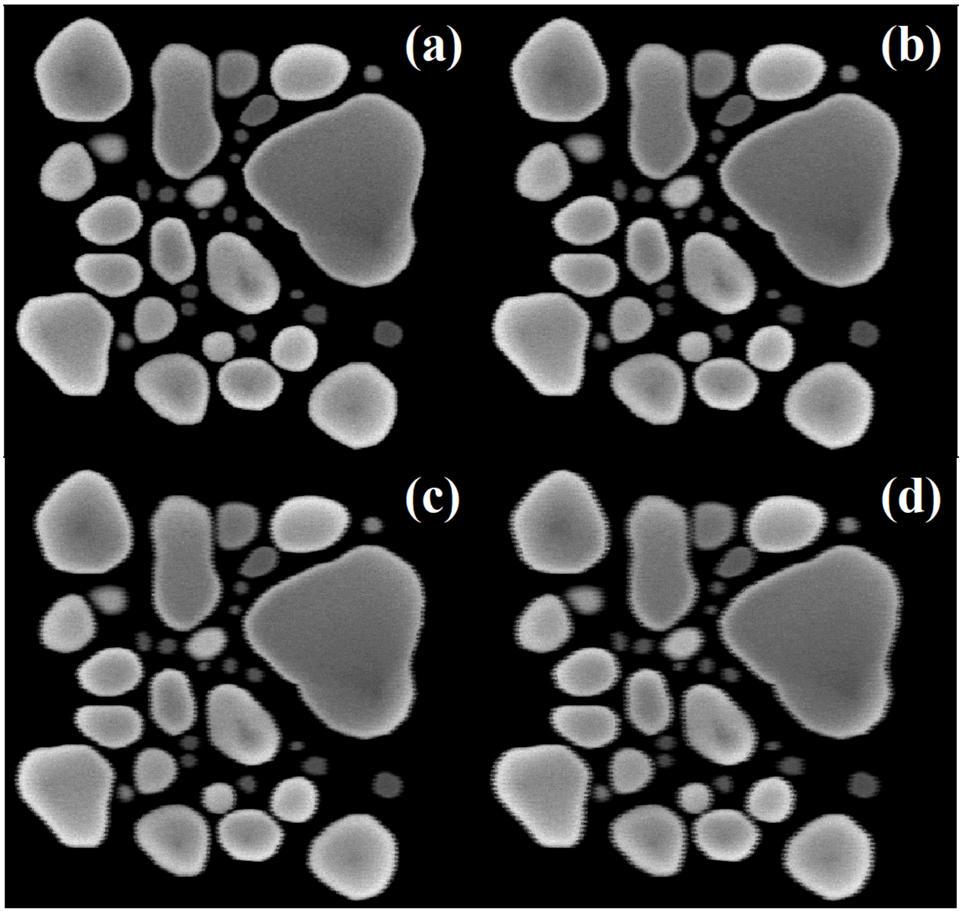

Figure S15 Simulated SEM images under different vibration amplitudes: (a) $A = 0.5$, (b) $A = 1.0$, (c) $A = 1.5$, and (d) $A = 2.0$.

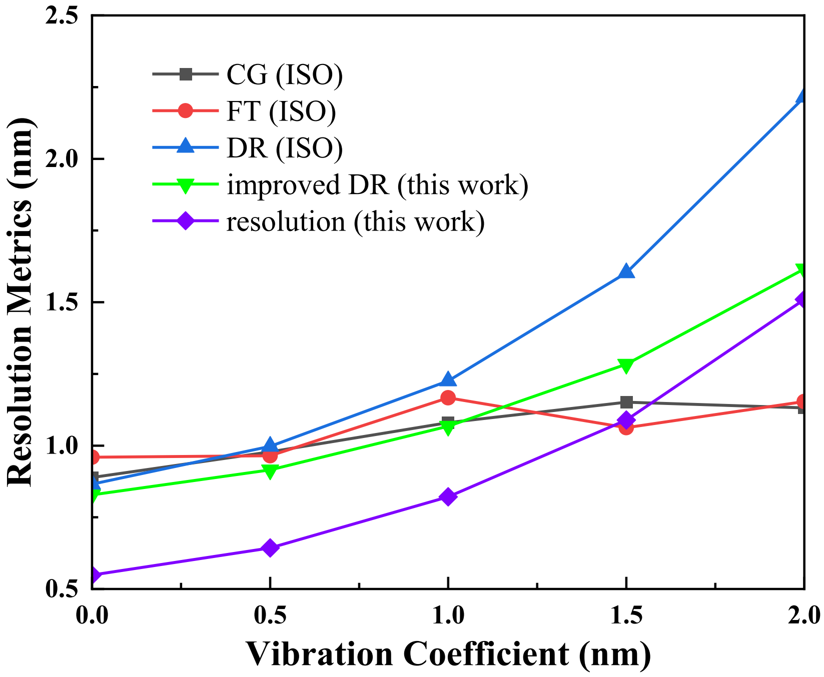

Figure S16 Sharpness and resolution of the processed SEM images in Figure S15 as functions of the vibration amplitude A.

Poisson noise can be introduced through Eq. (12). Figure S17 shows simulated SEM images under different numbers of incident electrons, N_e; the corresponding sharpness and resolution results are presented in Figure S18. As N_e decreases, the Poisson noise gradually increases. The sharpness values obtained by the original DR method are insensitive to Poisson noise, the CG method yields an unreasonable trend while the FT method presents an erroneously lower sharpness in case of strong Poisson noise ($N_e = 100$). The sharpness and resolution obtained by the improved DR method both increase with increasing Poisson noise, giving the correct variation trend.

According to Eq. (13), simulated SEM images under different Gaussian noise intensities σ_n are generated from Figure S10(c), as shown in Figure S19, and the corresponding sharpness and resolution results are presented in Figure S20. It can be seen that the sharpness values obtained by the FT, CG and original DR methods are likewise insensitive to the Gaussian noise intensity. The sharpness obtained by the improved DR method increases with increasing Gaussian noise, while the resolution, owing to the introduction of the Rose criterion, exhibits the correct and more pronounced variation trend.

In summary, under the systematic tests of different experimental factors, the sharpness values obtained by the CG, FT, and original DR methods either fail in some cases to reflect changes in image quality and may even yield incorrect trends or are insensitive to the factors. In contrast, the improved DR method and the resolution-based evaluation results provide consistent and physically reasonable variation trends under all these test conditions, thereby verifying the reliability and robustness of the present SEM image resolution evaluation method.

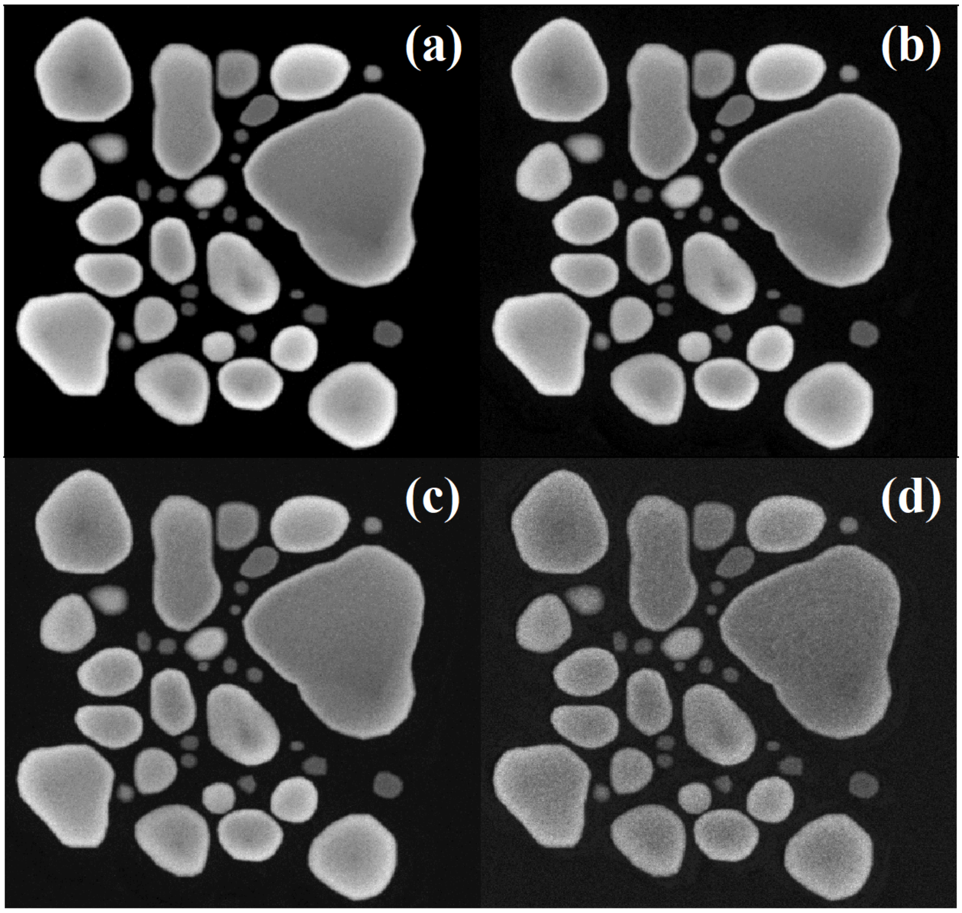

Figure S17 Simulated SEM images under different Poisson noise levels: (a) $N_e = 1600$, (b) $N_e = 900$, (c) $N_e = 400$, and (d) $N_e = 100$.

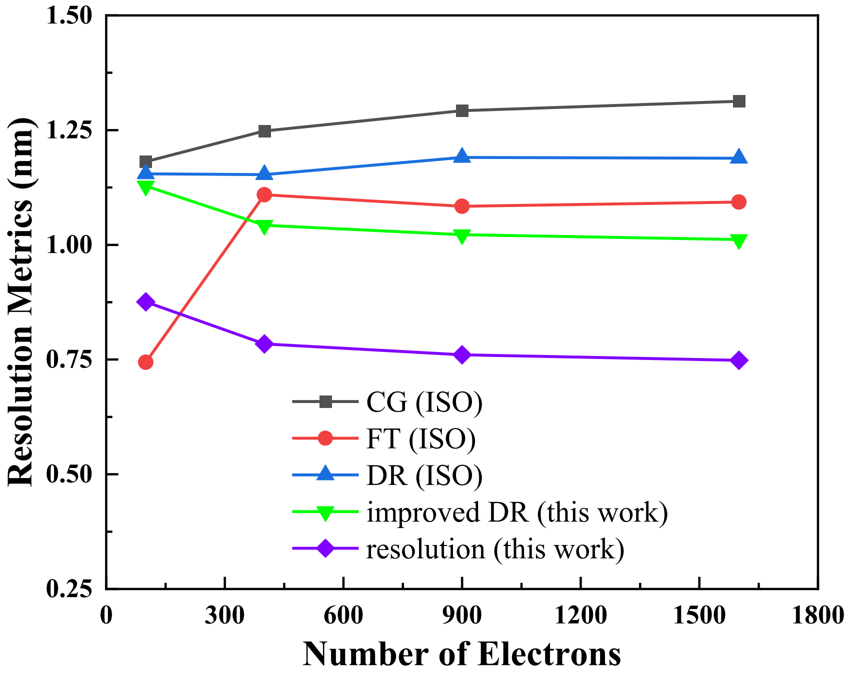

Figure S18 Sharpness and resolution of the processed SEM images in Figure S17 as functions of the number of incident electrons N_e.

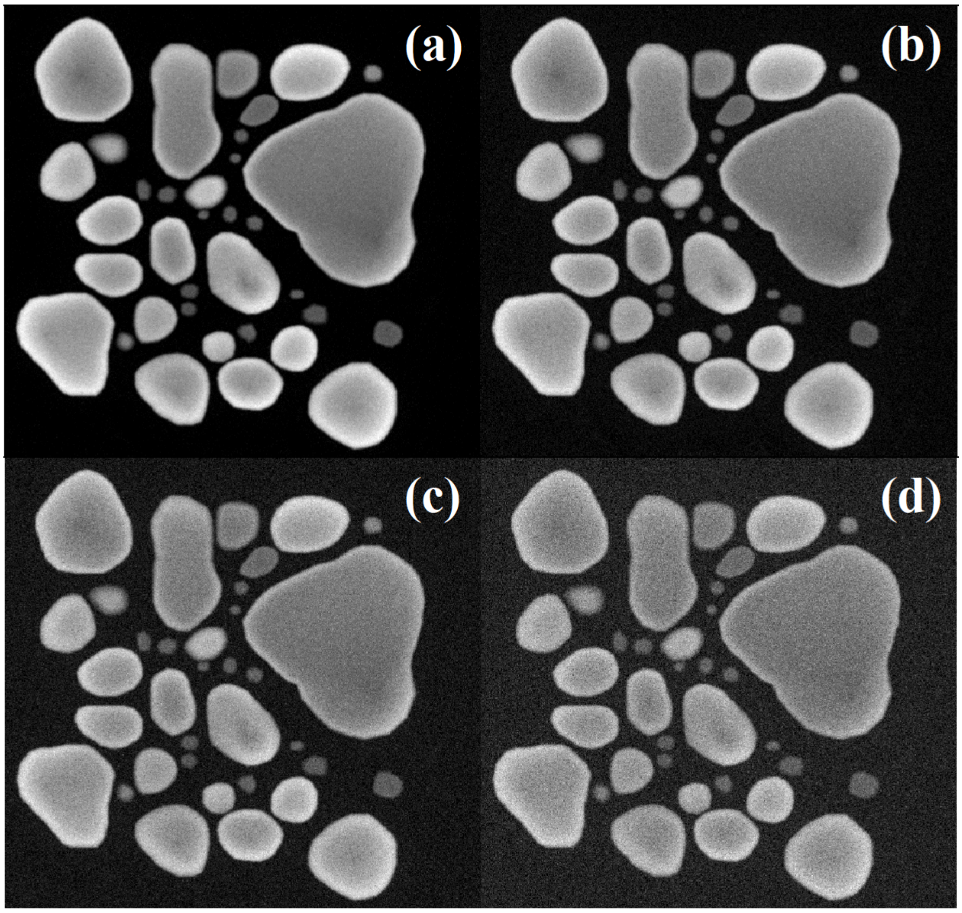

Figure S19 Simulated SEM images under different Gaussian noise intensities: (a) $\sigma_n = 4$, (b) $\sigma_n = 8$, (c) $\sigma_n = 12$, and (d) $\sigma_n = 16$.

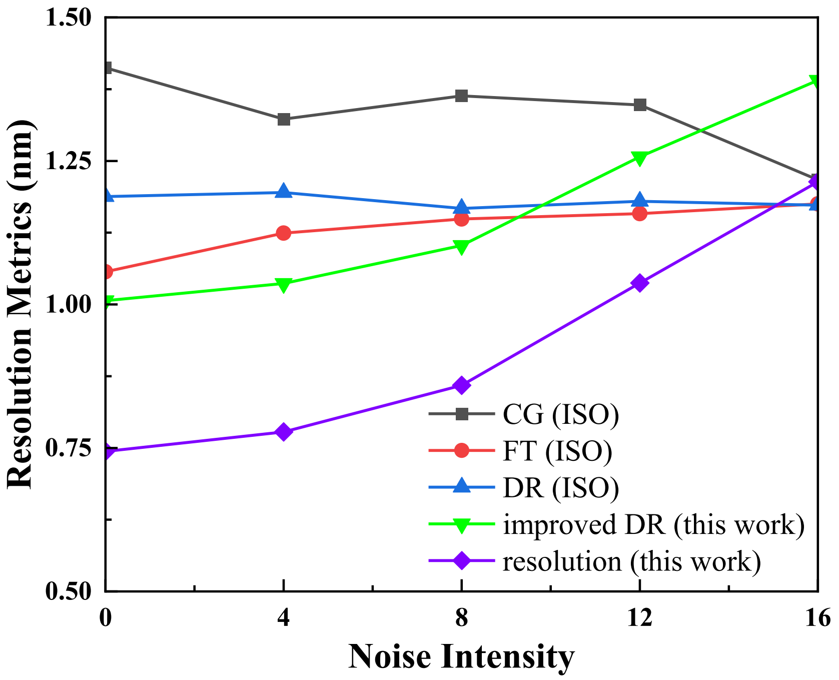

Figure S20 Sharpness and resolution of the processed SEM images in Figure S19 as functions of the Gaussian noise intensity σ_n.

S3. Uncertainty Analysis

This section aims to analyze the uncertainty of sharpness-resolution conversion curves due to the modeling of 3D morphology of gold particles. Figure S21 shows an experimental SEM image of Au/C sample acquired at an inclined incident condition for incident angle of 50° and at accelerating voltage of 15 kV. It can be seen that the gold particles are drum-shaped with flat top surface. In addition, larger particles generally exhibit smaller aspect ratios. Accordingly, we designed the particle structure configuration as shown in Fig. S22 for Monte Carlo simulation of beam-sample interaction. The particle diameter is denoted by *D*, the height of the curved cap by *h*, and the height of the cylindrical part by *H*. The corresponding aspect ratio is thus $\eta = (H + 2h)/D$. For particles with a diameter of 200 nm, the aspect ratio was set to 0.3, corresponding to $H = 0$ and $h = 0.15D$. For particles with a diameter of 5 nm, the aspect ratio was set to 0.75, corresponding to $H = 0.25D$ and $h = 0.25D$. For particles with diameters between these two limits, the geometric parameters were generated by linear interpolation.

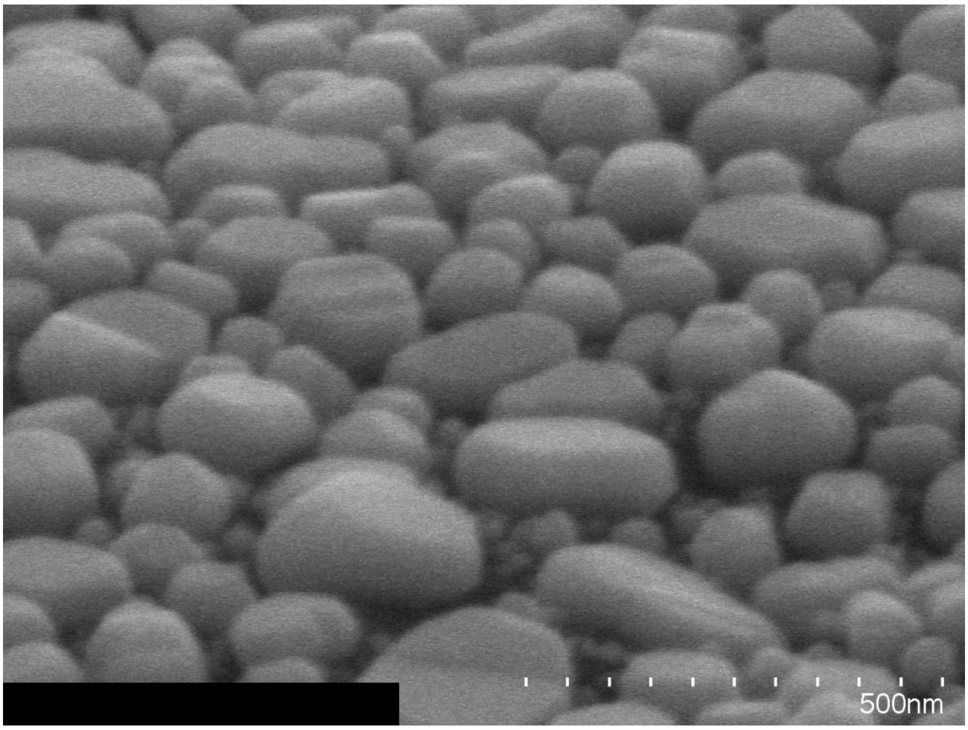

Figure S21 Experimental SEM image acquired at a tilt angle of 50° and accelerating voltage of 15 kV.

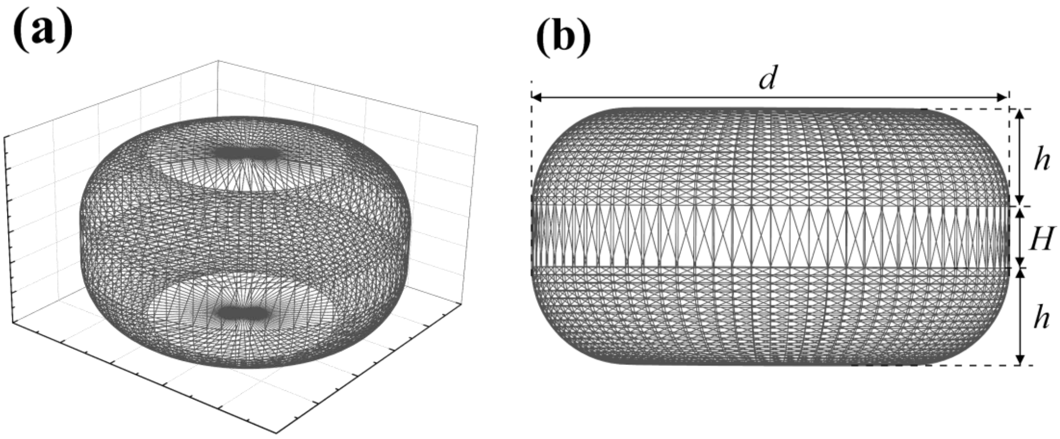

Figure S22 Schematic illustration of the particle geometrical structure model used in the present Monte Carlo simulation: (a) perspective view; (b) side view.

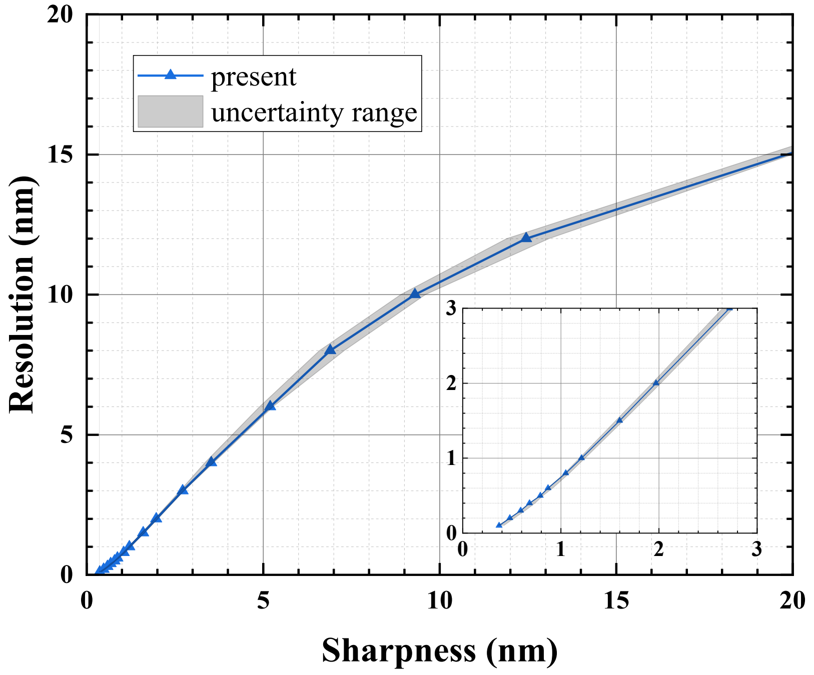

Figure S23 Sharpness-resolution conversion curve (blue line) together with the uncertainty range (gray shaded region) associated with the particle configuration for a particle diameter of 60 nm at an incident electron energy of 15 keV.

To estimate the uncertainty in the conversion curves arising from variations in particle configuration, we selected particles with a diameter of $D = 60$ nm and calculated several configurations with aspect ratios ranging from 0.4 to 0.7. The corresponding uncertainty range of the conversion curves was then obtained, as shown in Figure S23. The blue line represents the conversion curve adopted in the main text, while the gray shaded region indicates the uncertainty range. It can be seen that the uncertainty range increases very slightly with

increasing image sharpness. Nevertheless, the overall uncertainty remains quite limited and negligible. The reason for this is mostly related to the fact that the SE emission depth is very narrow in the order of nanometer. Then the SE intensity is almost independent of particle height, while the edge effect has a weak dependence on particle width. Therefore, the aspect ratio has negligible impact on conversion curve.

S4. Detector Type

This section discusses the applicability of the proposed method to the out-lens detectors with which the signal component changes from that of in-lens detector (ILD) (or through-the-lens detector) [4] and related issues to be considered. There are two types of commonly used out-lens detectors, backscattered electron (BSE) detector and Everhart-Thornley detector (ETD), in commercial SEMs. BSE emission has strong directional dependence, and the signals detected by a backscattered electron detector rely on its relative configuration with sample, which varies with the specific instrumental set-up and working distance. The present sharpness-resolution conversion method can in principle be extended to BSE detector to derive the resolution of BSE image by including detector geometrical factor. However, the tiny gold particles are less visible in a BSE image as compared to SE image. Moreover, in a BSE image the sharpness has strong dependence on the particle height as compared to the weak dependence on the particle width in an SE image. Because the sizes of particles in a resolution sample can differ significantly, the measured mean sharpness of BSE image will accompany a large uncertainty. Therefore, the resolution test of SEM instrument has been conventionally carried out with SE signals. The scope of the present work is thus limited to resolution evaluation of SE images.

The SE signals detected vary with the detector type. Those emitted from the sample directly by the incident beam are classified as SE1 and SE2, where SE1 are produced by the incident electrons at the immediate vicinity of the beam impact position and SE2 are produced by BSEs during their course of travel to the surface. In addition, BSEs can also generate remote SE emission when they strike the objective lens, the chamber wall and other component surfaces.

These strayed electrons are defined as SE3, which basically represent the BSE signal intensity but modulated by the local surface morphology.

An ILD detects merely SE1 and SE2 [4,5]. The SE yields for the different beam incident locations onto the *xy*-plane of the resolution sample are then calculated to derive the intensity map, or the simulated ideal SEM image over the sample projection plane, I_o in Eq. (7).

In comparison, for an ordinary ETD located below the electron optical column and by one side of sample, SE3 can also be detected as SE signals by the applied electric field (a small fraction of BSE detected is negligible). Evaluation of instrument resolution is usually carried out with ILD for a field emission SEM. However, ordinary SEMs (with W-filament electron source) and desktop SEMs use ETD as SE detector for which the necessary modification to the method is required. The problem arises because the exact portion of SE3, which can lead to changes in the imaging quality and the evaluated resolution, in total SE signal intensity depends on many instrumental factors (accelerating voltage, sample property, working distance, detector voltage, detector configuration etc.). A quantitative measurement has been reported for the gold particle in Au/C sample that the fraction of SE3 occupies ~61% of the total intensity [6].

Here we adopt an approximate algorithm to derive the likely image signal composition for an ETD detector. In a Monte Carlo simulation of beam-sample interaction, the information of a backscattered electron (energy E and emission angle θ) leaving the sample surface is recorded. It will strike the pole-piece surface if the emission angle falls within a specified angle θ_c, which is related to the size of pole-piece and working distance. It may generate SE3 there. Instead of performing another Monte Carlo calculation, here we use the experimental SE yield curve $\delta(E)$ of iron to estimate the SE3 intensity from the pole-piece surface as $\delta(E)/\cos\theta$, where the incident angle of backscattered electron onto the pole-piece is taken as θ by neglecting the local surface morphology. The total SE3 contribution is then obtained by summing over all such backscattered electrons simulated. Accordingly, the total number of detected signal electrons is written as

$$I_o = I_{SE1} + I_{SE2} + \alpha I_{SE3}, \qquad \text{(S6)}$$

where I_{SE1}, I_{SE2}, and I_{SE3} denote the signal intensities contributed by SE1, SE2, and estimated SE3 components, respectively. For an ILD, $I_o = I_{SE1} + I_{SE2}$. The coefficient α represents the collection efficiency of SE3 relative to that of SE1 and SE2 by an ETD. In a specific imaging measurement α is approximately constant and independent of scanning position. Therefore, α acts as a parameter for image simulation. By varying α values, the uncertainty range introduced by detector type into the sharpness-resolution conversion curve could be evaluated. However, here we use another parameter, f, the portion of SE3 among the total signals instead of using α. This is because the experimentally measured value $f \sim 60\%$ [6] is a good estimation of the average, and we can then derive the uncertainty range of the sharpness-resolution conversion curve for $\Delta f = 20\%$ through the simulation. The correspondence between α and f can also be obtained.

Figure S24 shows an example sharpness-resolution conversion curve established for ETD, where the single particle diameter of $d = 60$ nm is considered, the fraction of SE3 in the total SE signals was set to $f = 60\%$ (corresponding to $\alpha = 1.77$), and the critical angle was taken as $\theta_c = 75^\circ$. Furthermore, considering that f may vary among different SEM instruments or measurement conditions, the uncertainty range of the conversion curve was calculated for $\Delta f = 20\%$, as indicated by the gray shaded region in Fig. S24. For SEM images acquired using an ETD detector, the sharpness value is typically greater than 2 nm and the uncertainty range in this regime is relatively large, indicating that the SE3 contribution can have a significant influence on the resolution evaluation of SEM images acquired with an ETD. Here we note that the local surface morphology of pole-piece has potential influence on the conversion curve through a random modulation of SE3 intensity, which is expected to weaken the effect on sharpness by BSE and warrants a further investigation. Therefore, the method is recommended to apply with the understanding of the potentially large uncertainty at the moment.

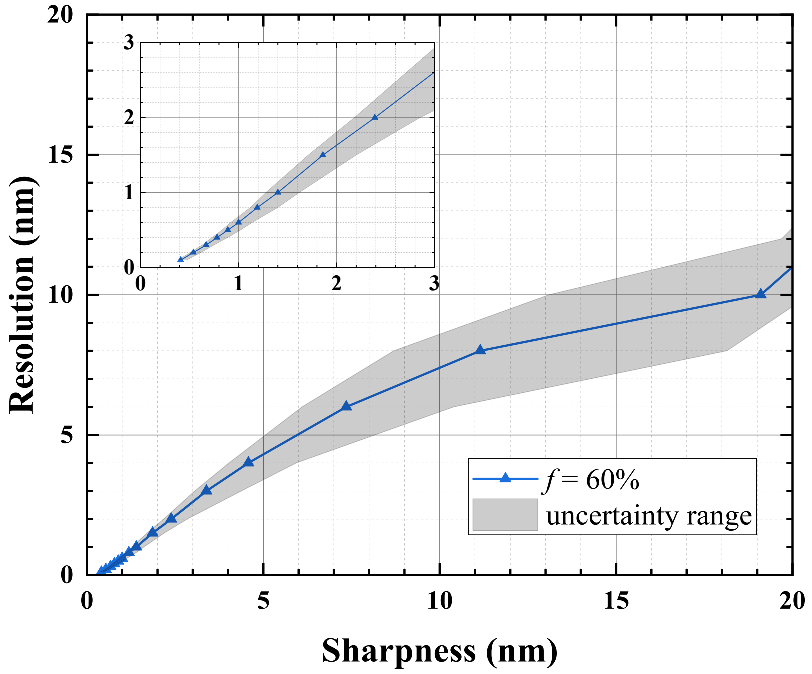

Figure S24 Sharpness-resolution conversion curve for ETD and for particle diameter of $d = 60$ nm, primary energy of 15 keV and $\theta_c = 75°$. The SE3 fraction is $f = 60\%$ ($\alpha = 1.77$) (blue line). The gray shaded region indicates the uncertainty range with the boundaries determined by $f = 40\%$ ($\alpha = 0.79$) and $f = 80\%$ ($\alpha = 4.69$).

S5. Supplementary Results

This section provides details for resolution tests, where part of the image data was taken from the SEM Resolution Test Program, Chinese Society for Testing and Materials (CSTM). To investigate the influence of noise on the resolution assessment scheme, the region of interest selected in Fig. 4 was subjected to three iterations of 3×3 median filtering, yielding the image shown in Fig. S25(a), which is at a noise level of 1.46. Subsequently, varying noise levels were introduced to produce the images in Figs. S25(b)-S25(f), corresponding to noise levels of 6.90, 13.41, 17.45, 21.43 and 25.34, respectively.

It is necessary to mention that in the application of the Rose-criterion-based conversion curve, the noise level should be evaluated in relative to the image contrast rather than solely by its absolute gray-value fluctuation. This is because the same absolute noise intensity may correspond to different effective noise levels for SEM images with different contrasts. To eliminate the influence of image contrast on the noise evaluation, the measured absolute noise intensity N is reduced by the image contrast C of the median-filtered image, and the

corresponding relative noise intensity is defined as $N_{\mathrm{rel}} = N/C$. In essence, the relative noise intensity is the reciprocal of the image contrast-to-noise ratio. The Rose resolution is then obtained by matching this relative noise intensity to the corresponding Rose conversion curve. For simplicity and clarity, the noise level is still described elsewhere in this work using the absolute noise intensity *N*, as defined in Eq. (17), with a default SEM image contrast of 175.

Sharpness was computed for each noisy image and then converted into Rayleigh and Rose resolutions, with the resulting trends plotted against noise intensity in Figure S26. As noise increases, all three metrics—sharpness, Rayleigh resolution, and Rose resolution—exhibit upward trends. Notably, Rose resolution increases much more rapidly than the other two, reflecting its stronger sensitivity to the noise. In principle, sharpness calculations should be independent of noise; however, in practice, noise affects critical stages of sharpness evaluation, such as binarization, edge detection, and edge curve fitting, thereby introducing deviations. This further affects the Rayleigh criterion resolution obtained by sharpness conversion. When noise becomes non-negligible, resolution is expected to increase significantly with noise level. Therefore, our hybrid resolution evaluation strategy, which incorporates both the Rayleigh and Rose criteria, offers a rational approach to account for both optical and noise-limited regimes. A critical threshold is observed at the noise level of $N = 13.41$, where the Rayleigh and the Rose resolutions converge. For $N < 13.41$, the Rayleigh resolution is adopted as the evaluation value, whereas for $N > 13.41$, the Rose resolution becomes effective.

Figure S27-S33 show ILD detected SEM images of different Au/C samples acquired at different magnifications and with different SEM instruments. For all of these images, an 800×800-pixel or 600×600-pixel region (indicated by the red box) cropped from an original SEM image was used for the calculation of sharpness and resolution, and the results are summarized in Table S1. Here, R_{FT} and R_{DR} denote the image sharpness values obtained by the improved FT method and the improved DR method, respectively, whereas R_{DR} (ISO) denotes the image sharpness values obtained by the original DR method presented in ISO/TS 24597 [1]. The final resolution $\mathfrak{R}$ is evaluated according to Eq. (18), rounding to one decimal place. Under the normal circumstances, the noise level in SEM images is low, and the final resolution

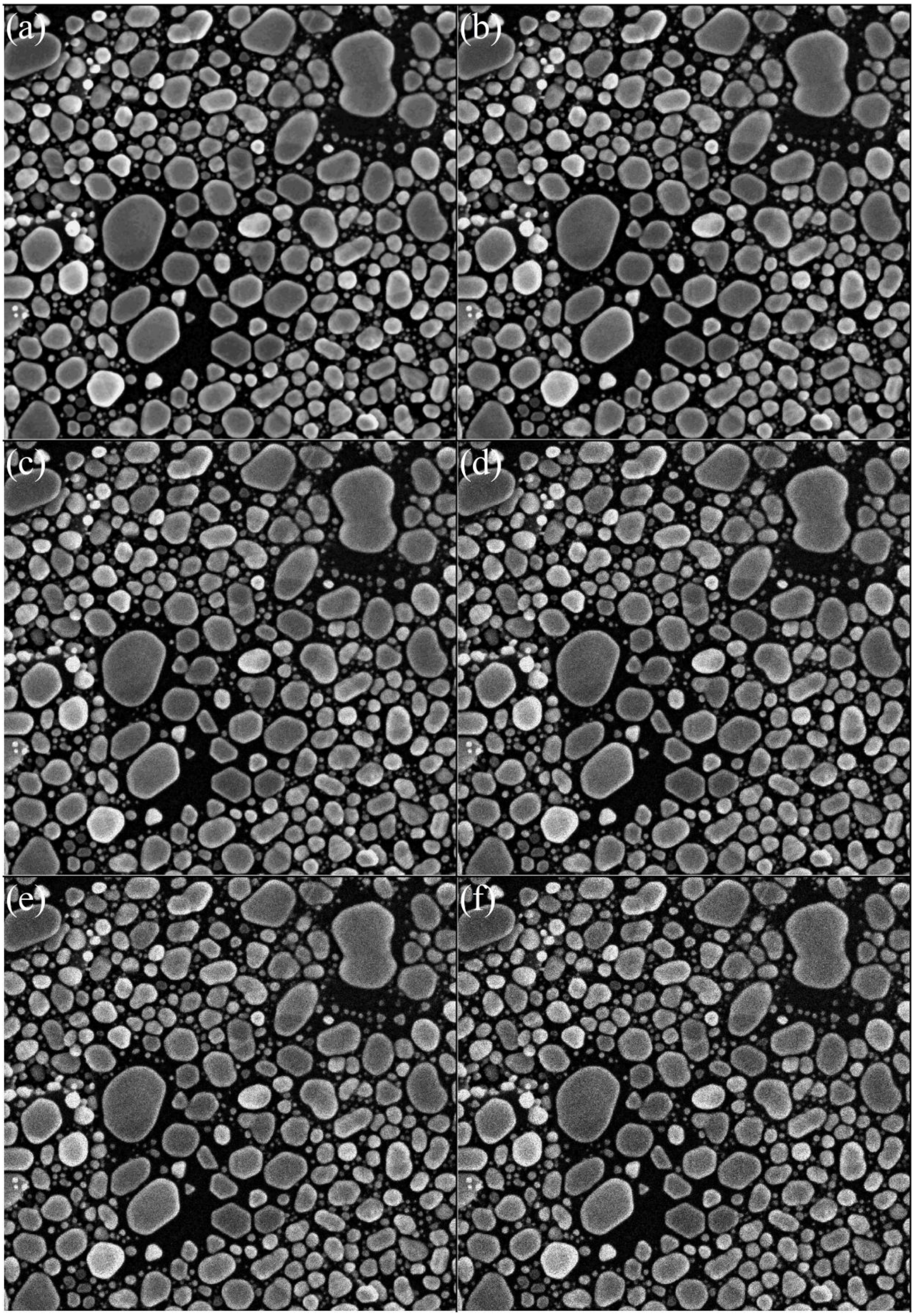
(a)
(b)
(c)
(d)
(e)
(f)

Figure S25 (a) Image obtained from the region of interest in Fig. 4 by applying triple median filtering, with a noise intensity of 1.46. (b-f) Images derived from (a) by progressively increasing the noise level to: (b) 6.90, (c) 13.41, (d) 17.45, (e) 21.43, (f) 25.34.

$\mathfrak{R}$ is limited only by the Rayleigh criterion. When obvious noise is present in the image, however, as in Fig. S28, the image resolution is then limited by the Rose criterion. Figures S29-S31 are measured by the same instrument at three different magnifications. Similarly, Figures S32-S33 are measured by another instrument at two different magnifications. At two higher magnifications, the changes in the determined $\mathfrak{R}_{Rayleigh}$ are merely ~3.1% and 0.2% for the involved two instruments, while the changes in R by the improved DR method are 7.2% and 3.4% and the changes in R by the ISO-defined DR method are ~26.4% and 18.6%, respectively. Particularly, R_{DR} (ISO) tends to be smaller at higher magnifications but $\mathfrak{R}$ is rather stable. This fact indicates that the resolution $\mathfrak{R}$ is a much better metric than sharpness R for representing instrumental capability, i.e. the resolving power.

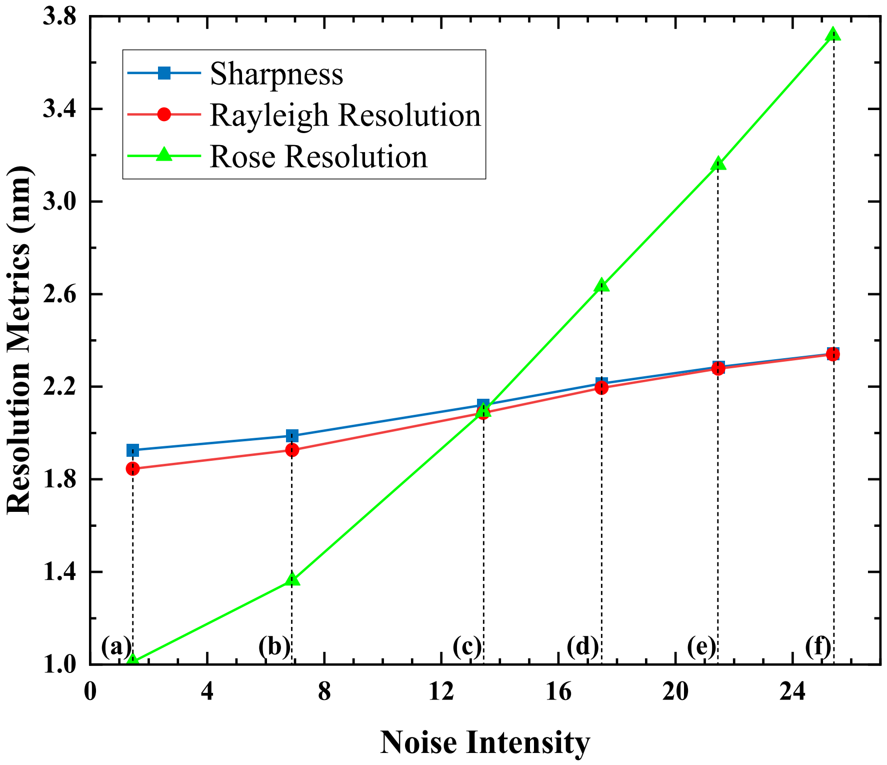

Figure S26 Trends of sharpness, Rayleigh resolution and Rose resolution as functions of noise intensity, evaluated from

Figure S25. Points (a)-(f) on the horizontal axis correspond to the noise levels applied in

Figure S25(a)-S25(f).

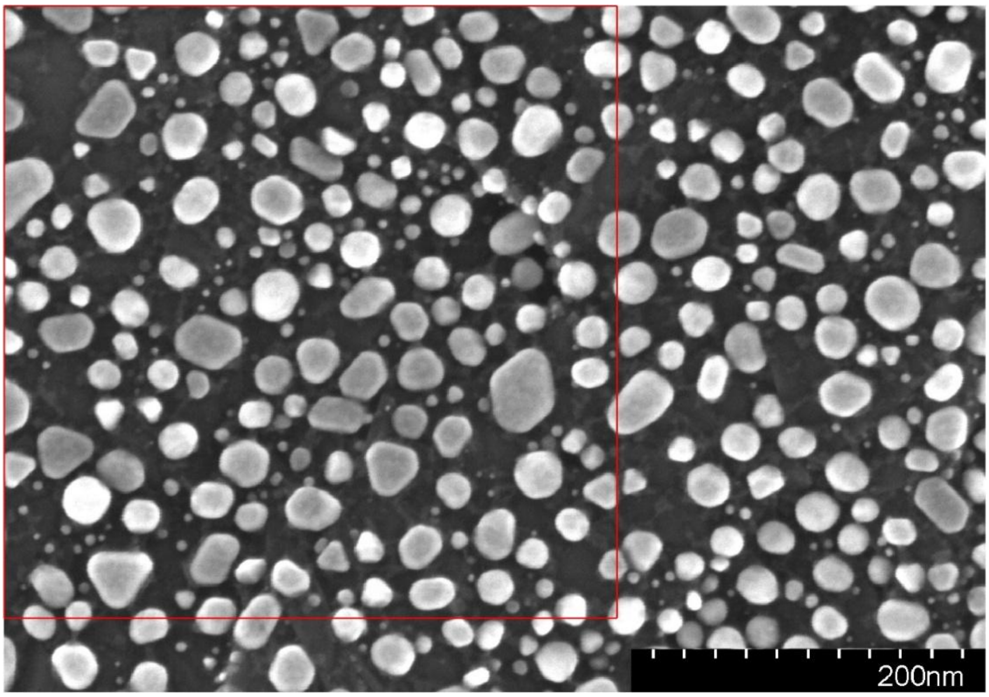

Figure S27 An 800×800-pixel region (red box) cropped from an SEM image of an Au/C sample for the calculation of sharpness and resolution. The accelerating voltage is 15 kV, and the magnification is 200,000×.

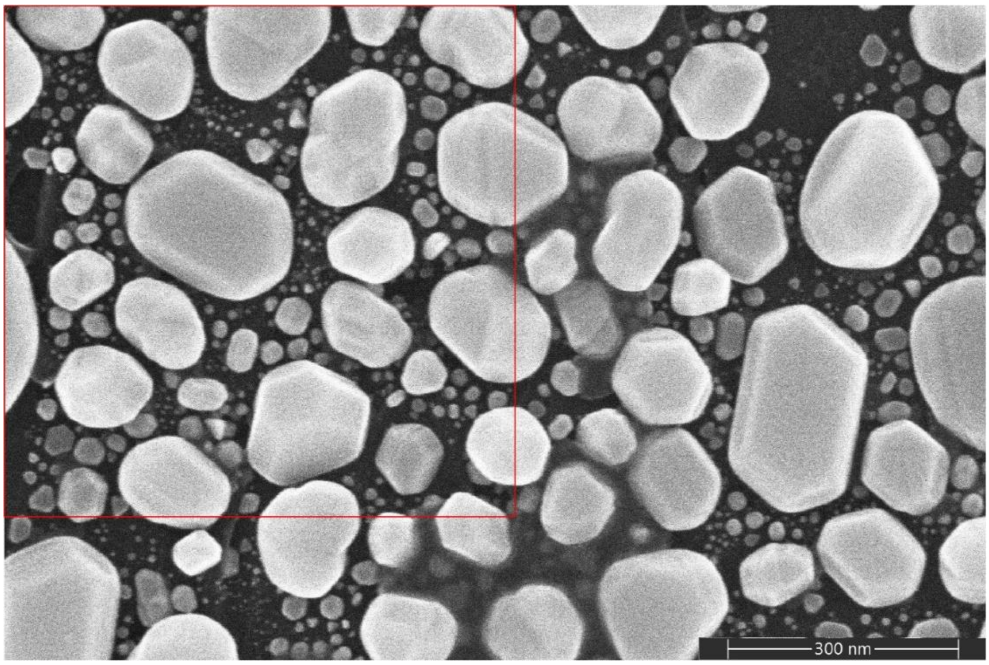

Figure S28 An 800×800-pixel region (red box) cropped from an SEM image of an Au/C sample for the calculation of sharpness and resolution. Obvious noise can be observed in this image. The accelerating voltage is 15 kV, and the magnification is 100,000×.

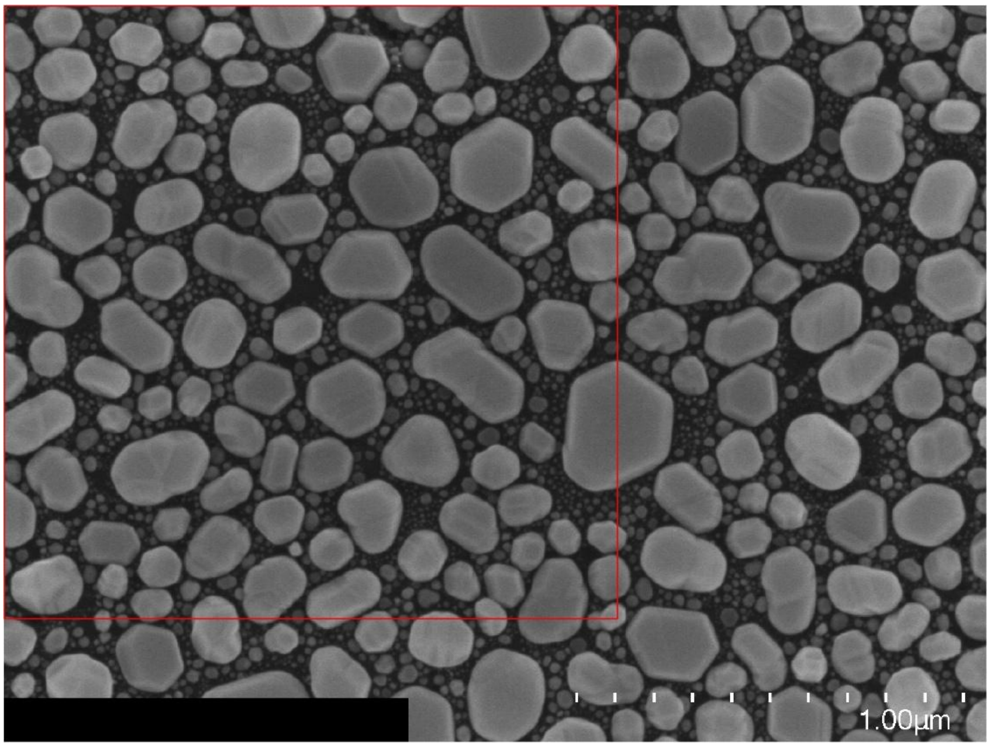

Figure S29 An 800×800-pixel region (red box) cropped from an SEM image of an Au/C sample for the calculation of sharpness and resolution. The accelerating voltage is 15 kV, and the magnification is 50,000×.

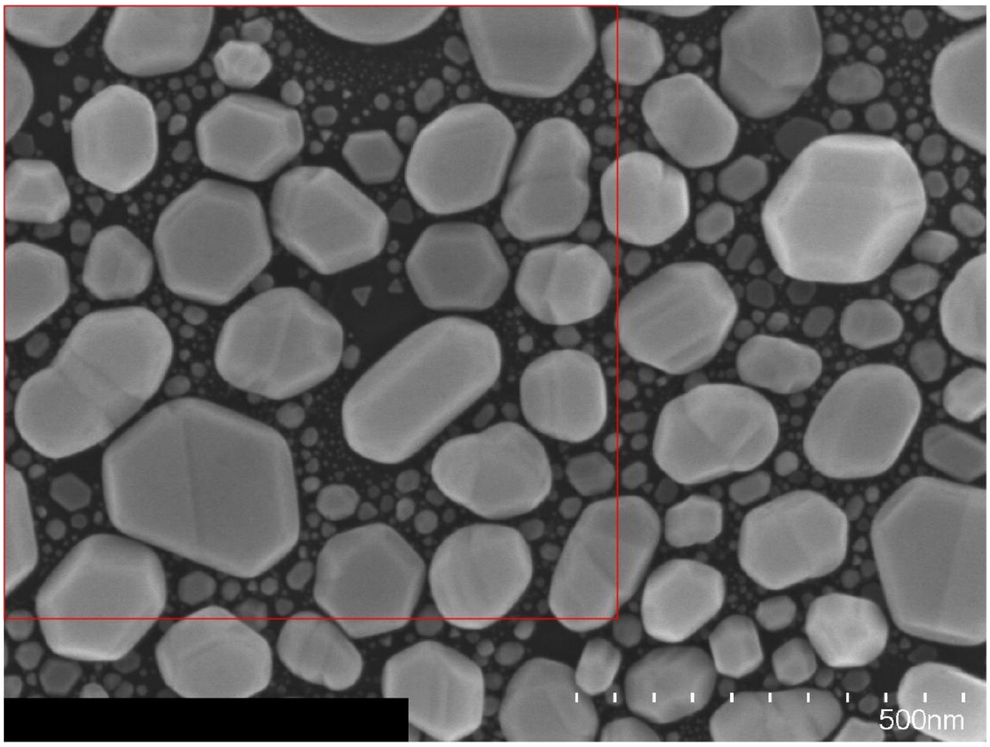

Figure S30 An 800×800-pixel region (red box) cropped from an SEM image of an Au/C sample (same as that of Fig. S29) for the calculation of sharpness and resolution. The accelerating voltage is 15 kV, and the magnification is 100,000×.

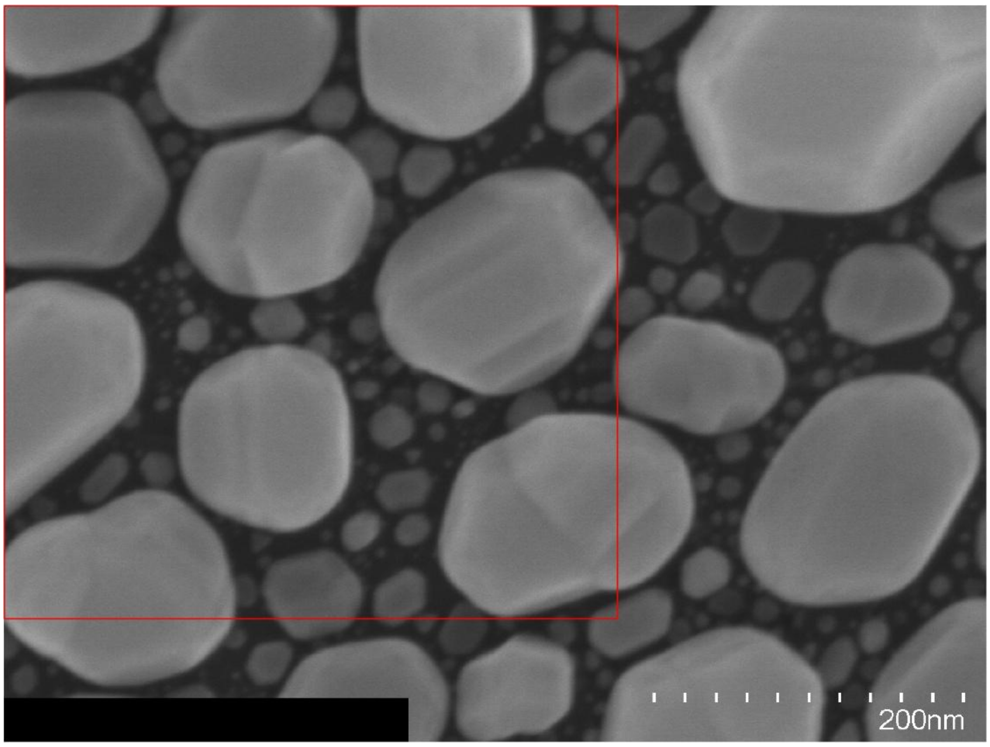

Figure S31 An 800×800-pixel region (red box) cropped from an SEM image of an Au/C sample for the calculation of sharpness and resolution. The accelerating voltage is 15 kV, and the magnification is 200,000×. The imaging is taken for an area in Fig. S30.

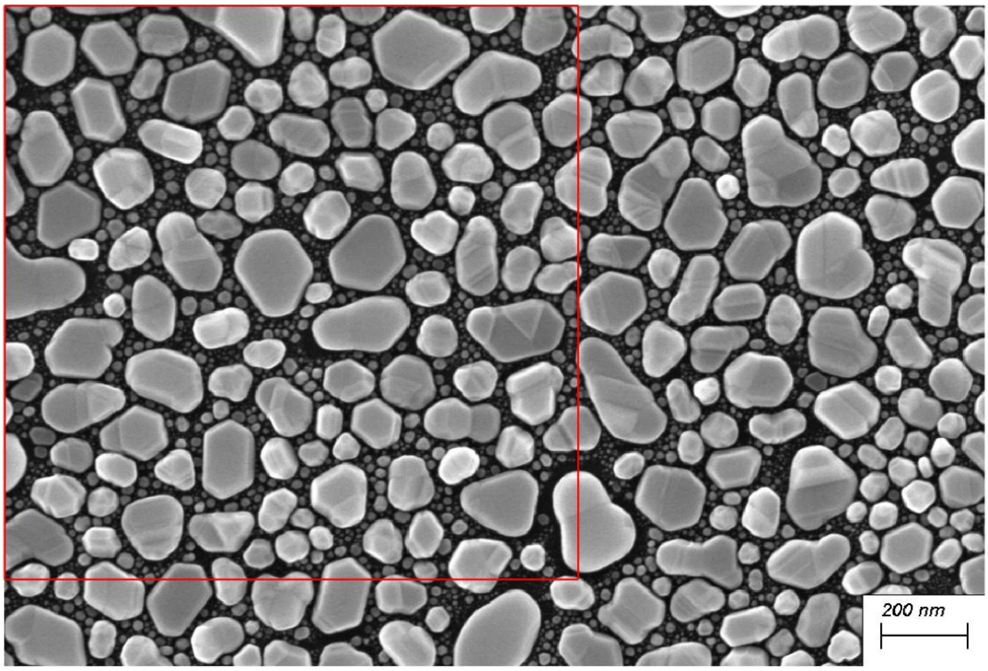

Figure S32 A 600×600-pixel region (red box) cropped from an SEM image of an Au/C sample for the calculation of sharpness and resolution. The accelerating voltage is 15 kV, and the magnification is 50,000×.

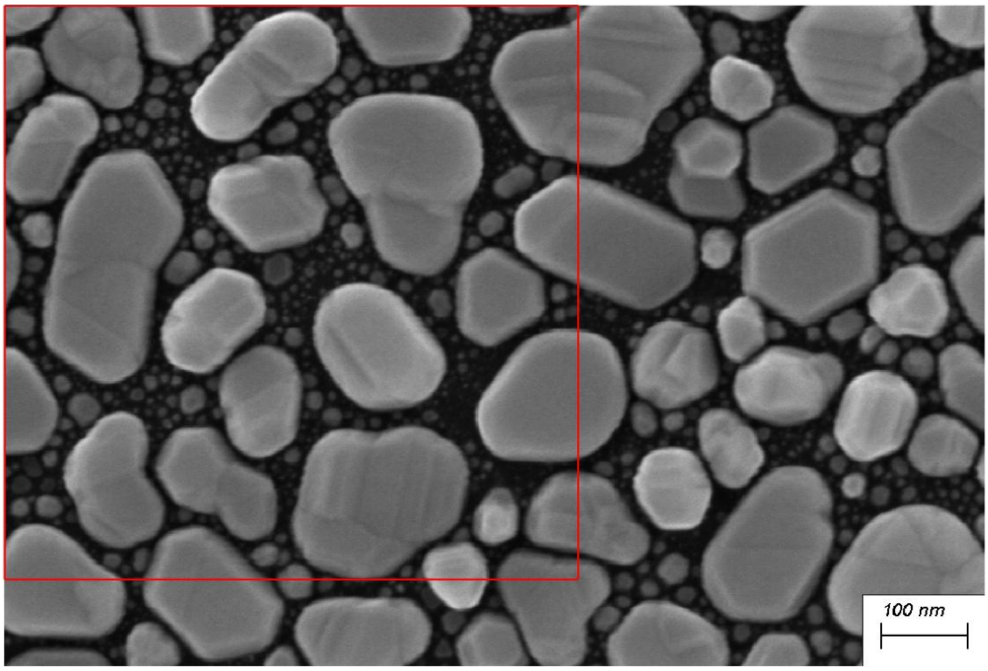

Figure S33 A 600×600-pixel region (red box) cropped from an SEM image of an Au/C sample for the calculation of sharpness and resolution. The accelerating voltage is 15 kV, and the magnification is 100,000×.

Table S1. Evaluation results of sharpness and resolution for SEM images of Au/C samples

figure	pixel size (nm/pixel)	R_{FT} (nm)	R_{DR} (ISO) (nm)	R_{DR} (nm)	$\mathfrak{R}_{\mathrm{Rayleigh}}$ (nm)	$\mathfrak{R}_{\mathrm{Rose}}$ (nm)	$\mathfrak{R}$ (nm)
Fig. S27	0.496	1.140	1.606	1.459	1.268	0.740	1.3
Fig. S28	0.833	1.918	2.492	2.463	2.422	2.851	2.9
Fig. S29	1.984	4.325	5.053	5.176	5.431	3.632	5.4
Fig. S30	0.992	2.556	3.452	3.235	3.163	1.962	3.2
Fig. S31	0.496	3.108	2.731	3.469	3.262	2.060	3.3
Fig. S32	2.222	3.697	4.691	3.471	3.583	2.325	3.6
Fig. S33	1.111	2.512	3.955	3.588	3.577	2.199	3.6

Supplementary References

[1] *ISO/TS 24597:2011 Microbeam analysis–Scanning electron microscopy–Methods of evaluating image sharpness*. ISO: Geneva, Switzerland (2011).

[2] https://www.jeolusa.com/RESOURCES/Electron-Optics/Documents-Downloads/resolution-in-sem

[3] P. Zhang, H.Y. Wang, Y.G. Li, S.F. Mao and Z.J. Ding. *Monte Carlo simulation of secondary electron images for real sample structures in scanning electron microscopy*. Scanning 34(3) (2012): 145-150.

[4] J.I. Goldstein, D.E. Newbury, J.R. Michael, N.W.M. Ritchie, J.H.J. Scott and D.C. Joy. *Scanning Electron Microscopy and X-Ray Microanalysis* (4th Ed.). Springer (2018).

[5] H.T. Chen, Y.B. Zou, B. Da and Z.J. Ding. *A Quest for the mechanism of ultrahigh resolution SEM imaging*. Advanced Science 13 (2026): e16341.

[6] K.-R. Peters. *Generation, collection and properties of an SE-I enriched signal suitable for high resolution SEM on bulk specimens*. Scanning Electron Microscopy 1982(1) (1982): 363-372.
